# A new class of transformable kirigami metamaterials for reconfigurable electromagnetic systems

**DOI:** 10.1038/s41598-022-27291-8

**Published:** 2023-01-21

**Authors:** Yunfang Yang, Andrea Vallecchi, Ekaterina Shamonina, Christopher J. Stevens, Zhong You

**Affiliations:** Department of Engineering Science, University of Oxford, Parks Road, Oxford, OX1 3PJ UK

**Keywords:** Electrical and electronic engineering, Optomechanics, Design, synthesis and processing, Surface patterning, Mechanical engineering, Materials for devices, Structural materials, Techniques and instrumentation

## Abstract

The rapid development of radio frequency (RF) components requires smart multifunctional materials that can adapt their physical shapes and properties according to the environment. While most current reconfigurable systems provide limited flexibility with high manufacturing cost, this research proposes to harness the transformable properties of kirigami-inspired multistable mechanical metasurfaces that can repeatedly deform and lock into different configurations to realize a novel class of low-cost reconfigurable electromagnetic structures with a broad design space. The metasurfaces are formed by designing kinematic-based unit cells with metallised coating that can provide adjustable resonant electromagnetic (EM) properties while rotating with respect to each other. Tailoring the cut length and geometry parameters of the patterns, we demonstrate programming of the topologies and shapes of different configurations. The influence of critical parameters on the structural multistability is illustrated by means of both a simplified energy model and finite element simulations. As examples of the reconfigurable electromagnetic devices that can be realized, we report the development of a tuneable half-wave dipole and two frequency selective surface (FSS) designs featuring isotropic and anisotropic responses. While the kirigami dipole can be tuned by mechanically stretching its arms, the FSSs exhibit distinct transmittance and reflectance spectra in each of the kirigami patterns stable states. The functionality of these kirigami devices is validated both by full-wave EM simulations and experiments. The proposed transformable structures can be mechanically actuated to tune the EM response in frequency or induce anisotropies for wave propagation.

## Introduction

Expansion of wireless communication and the increasing diversity of advanced wireless services have resulted in a growing demand for reconfigurable electromagnetic (EM) systems capable of supporting seamless user mobility across different wireless access technologies. Among the key components to design multi-standard transmitter and receiver architectures are tuneable antennas and reconfigurable frequency selective surfaces (FSSs). In most tuneable antenna and FSS designs, reconfigurability is achieved by changing the current patterns in the antenna or unit cell of FSSs using switches, such as pin diodes and micro-electromechanical switches (MEMSs), or loading the structures with varactor diodes, which provide a variable voltage-controlled capacitance^1–4^. However, the biasing and control circuits required to drive these active components increase the complexity of the system, and can be the source of interference and reflections, besides introducing additional conduction losses, thereby contributing to the reduction of the overall performance, especially at high frequencies.

Recently, new approaches to tune the response of electromagnetic components based on mechanical transformation have been proposed. In the FSS composed of ceramic resonators with different band stop responses under front and side incidences developed in^5^, the response can be reconfigured between two adjacent stopbands by simply mechanically changing the orientation of the ceramic resonators. Particularly interesting is a family of mechanical metamaterials that can exploit shape morphing behaviour to tune their mechanical as well as dielectric properties^6–9^. Mechanical metamaterials with superior structural flexibility can have low electromagnetic loss for millimetre waves, while requiring comparatively low fabrication cost; this make them attractive candidates for the realization of reconfigurable electromagnetic components, which are crucial for many sectors, such as the next generations of wireless communications systems, 5G and beyond, supporting multi-mode and multi-band applications^10–12^, and thus requiring multi-function reconfigurable antennas to replace multiple of single function legacy antennas^13–16^. Reconfigurable sensors for the nondestructive remote extraction and monitoring of various quantities such as strain, dielectric material and liquid properties^17,18^, and wearable electronics^19^, where reshaping capabilities can increase the adaptability and compliance of the electronic platform to the human body and thus can be instrumental for biomedical technologies^20,21^.

Generally, mechanical reconfiguration can have some advantages with respect to electronic components, for example, it would not require high bias voltages, and thus no bias control circuits that can add complexity and losses to the system. Moreover, in mechanical metamaterials there are no spurious frequency components due to the non-linearity of active devices, and they lend themselves to high-power applications because there is no risk of an electrical breakdown. On the other side, the use of tunable materials such as ferroeletrics, ferrites, and liquid crystals, whose electrical properties can be controlled by the application of external bias (e.g. heat, electric or magnetic field, optical radiation, etc.), usually require high bias voltages or high DC power consumption and show unwanted sensitivity to thermal variations. As a possible drawback, mechanically reconfigurable RF devices tend to have relatively slower tuning response, which however can be improved through the use of suitable materials and manufacturing methods and specific implementations of the required external mechanical actuator to achieve faster deployment. Comprehensive reviews of the applications of different tuning techniques including mechanical actuation, tunable materials and integrated electronic devices, with benefits and disadvantages, can be found in^22,23^, with reference in particular to metasurfaces.

This research aims at leveraging the transformable properties of mechanical metamaterials to develop a novel class of reconfigurable electromagnetic devices. By designing kinematic-based unit cells that can rotate with respect to each other and equipping every unit with metallised elements to provide adjustable resonant electromagnetic properties, we are able to mechanically actuate the transformable structure to tune the EM response in frequency or induce anisotropies for wave propagation.

Origami-based structures have been previously suggested as a solution to create deployable continuous-state tuneable FSSs, in which an origami pattern enables the change in the overall shape of the structure that provides the desired reconfigurability^24–26^; yet, the out-of-plane deformation of origami structures makes their application difficult in compactly constructed planar components. Planar auxetic structures with buckling-induced elements have been also proposed for electromagnetic compatibility applications^27,28^. These kinematic mechanisms usually have continuous small deformation; therefore, they need precise control to deploy to the specific configurations that provide the desired variable frequency response. Achieving a robust and stable switching between several configurations is very challenging, and usually requires a pre-stressed state to be maintained in the structure^29^. To address this problem, we develop a multistable structure that can repeatedly lock into several configurations, with distinct EM responses. Multistable structures have multiple equilibrium states where the potential energy reaches local minima, so the structure would automatically tends to stay in these configurations, which gives them a significant advantage over continual motion structures for switchable materials^30,31^.

The multistable designs we developed in this work are inspired by kirigami art^32^. By tuning the cut length and geometry parameters of the patterns, we demonstrate programming of the topologies and shapes of different configurations. Both results based on a simplified energy model and finite element simulations are presented to illustrate the influence of critical parameters on the structural bistability. We show that these structures can be exploited to realize a variety of reconfigurable electromagnetic devices. In particular, we focus on the development of a tuneable half-wave dipole and two FSS designs, one of which exhibits an isotropic response while the other is anisotropic. Both the dipole and FSSs can be realized by creating an electrically conductive/metallic layer on one side of the rubber sheet onto which the kirigami pattern is cut; another option would be to form the kirigami structure with a high permittivity material, but in this work we focus on the former approach. Once covered with a suitable metallization, the kirigami dipole response can be tuned by mechanically stretching its arms, while the kirigami metasurfaces would behave as FSSs exhibiting specific transmittance and reflectance spectra in each of their stable states.

The paper has the following structure. “Results” section presents the simulations and experiments conducted to validate the functionality of proposed kirigami devices. In “Discussion and conclusions” section, the manufacturing approach is discussed and the concluding remarks are drawn, while details of structural energy analysis are delegated to the Supplementary Materials section.

## Results

### Structure design

We focus on kirigami patterns that have the following feature: (1) the closed state of the metamaterial has no voids on it so as to mimic a conductive screen; (2) the stable states of the metamaterials are planar structures so as to behave as planar frequency selective surfaces; (3) the metamaterial has bistable behaviours so it can lock in various stable states. Following this path, we take inspiration from kirigami geometric motifs and bistable structures design. We particularly focus on two designs: a triangle pattern that can elongate in a single direction, and a star pattern that can deploy orthotopically. By rotating the triangular element in the unit cells, the pattern can expand to multiple configurations and retain the deformation after the load is released.

#### Triangular pattern

**Figure 1:**
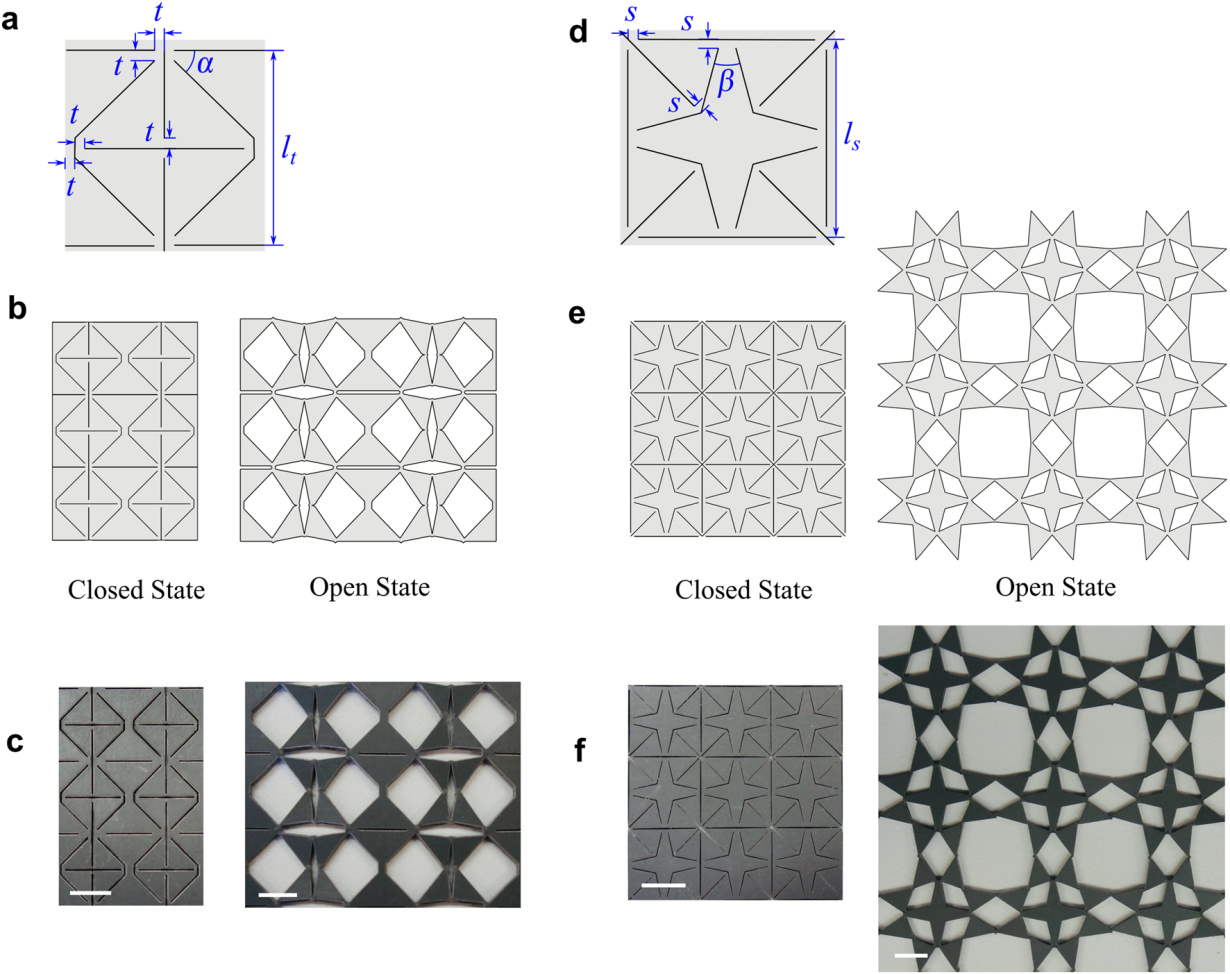
Metamaterial structure design. Design parameters of the unit with (**a**) triangular pattern and (**d**) star pattern. The close and open states of the tessellated (**b**) triangular metamaterial and (**e**) star metamaterial. Photograph of the laser-engraved sample with (**c**) triangular and (**d**) star patterns, in their close and open states. The length of the scale bar is 1 cm.

As shown in Fig. 1a, the unit of the triangle cutting pattern comprises eight triangles connected to each other at their vertices via thin ligaments. When the unit is stretched horizontally, the thin ligaments act as flexure hinges, and the triangles are capable of rotating about the connection vertices with respect to the adjacent ones. This deployment generates three quadrilateral voids inside the unit cell. When the unit cell is stretched to a specific configuration, it is able to lock in an open state. To recover the unit cell to its original configuration, a pair of compressive forces needs to be applied to the unit cell to enable it snap back to the close state. Tessellating the unit cell in both horizontal and vertical directions creates a metasurface. Figure 1b shows an example consisting of 2 × 3 units cells. When subjected to uniform stretching or compression horizontally, the unit cells in the same column will have equivalent motions because their width is constrained by the adjacent top and bottom units; on the other hand, in each row, the unit cells can lock into different configurations, leading to a multistable feature (Movie M1). In this way, we can generate metasurfaces with different topologies by deploying certain columns of the same structure (Movie M2). Figure 1c shows a metamaterial of the same pattern made by laser engraving a rubber sheet in its open and close states corresponding to those in Fig. 1b.

The deformation of the metamaterial can be modelled as an assembly of planar mechanisms. If we assume the thickness of the flexure hinges is zero, i.e., the triangles have an ideal vertex-to-vertex connection, the transformation of the structure can be regarded as a mechanical motion where all triangular pieces are rigid bodies (Supplementary Materials S2). However, in the real material deformation process, the structure does not have perfect hinges at the vertices. Instead, the flexure hinges are bent and unbent during the transformation, which leads to an elastic strain energy variation. This results in multiple stable states from the closed state to fully deployed open state. In other words, the metasheet is capable of staying in a number of deformed shapes even if the load is removed.

#### Star pattern

While the previous triangle pattern is able to elongate in a single direction, we further develop a star pattern which can deploy isotropically. As shown in Fig. 1d, the unit cell consists of eight small triangles connected by a four-point star. Kinematically, the structure is not able to deploy if no deformation in the star and triangles is permitted. However, if the dimensions of thin ligaments are carefully tailored, the unit cell is transformable through a snap-through process. A quarter of the unit cell is similar to the connection arrangement of the triangle pattern: during the deformation, the star restricts the distance between a pair of these triangles, so the two small triangles would compress the middle ligament severely to rotate outwards, leading to a snap-through behaviour. If we stretch a 3 × 3 metasheet uniformly along its four corners, it snaps into an isotropic open pattern as shown in Fig. 1e (Movie M1). If the force is applied along one of the diagonal directions, the structure forms a semi-open diamond shape where only two pairs of the triangles open, as shown in Supplementary Materials S2. While the semi-open state of the star pattern features an anisotropic response with different transmittance depending on the polarisation of the incoming wave, the closed and open patterns are substantially isotropic due to the four line and four-fold rotational symmetry of their unit cells. In this research, we particularly focus on the responses of the latter isotropic states.

When tessellated in the plane, the geometry of the unit cell is coupled with its neighbours in two directions, therefore, the number of the stable states remains the same regardless of the number of unit cells. Movie M2 demonstrates the reconfiguration process of the star metasurface. The structure is only geometrically compatible at the close state and open state. Figure 1f shows the laser cut prototype of a 3 × 3 unit star metasheet. Note that it may tend to have an out-of-plane deformation in the video when the structure was cut out of a rather thin rubber sheet.

### Bistability analysis

**Figure 2:**
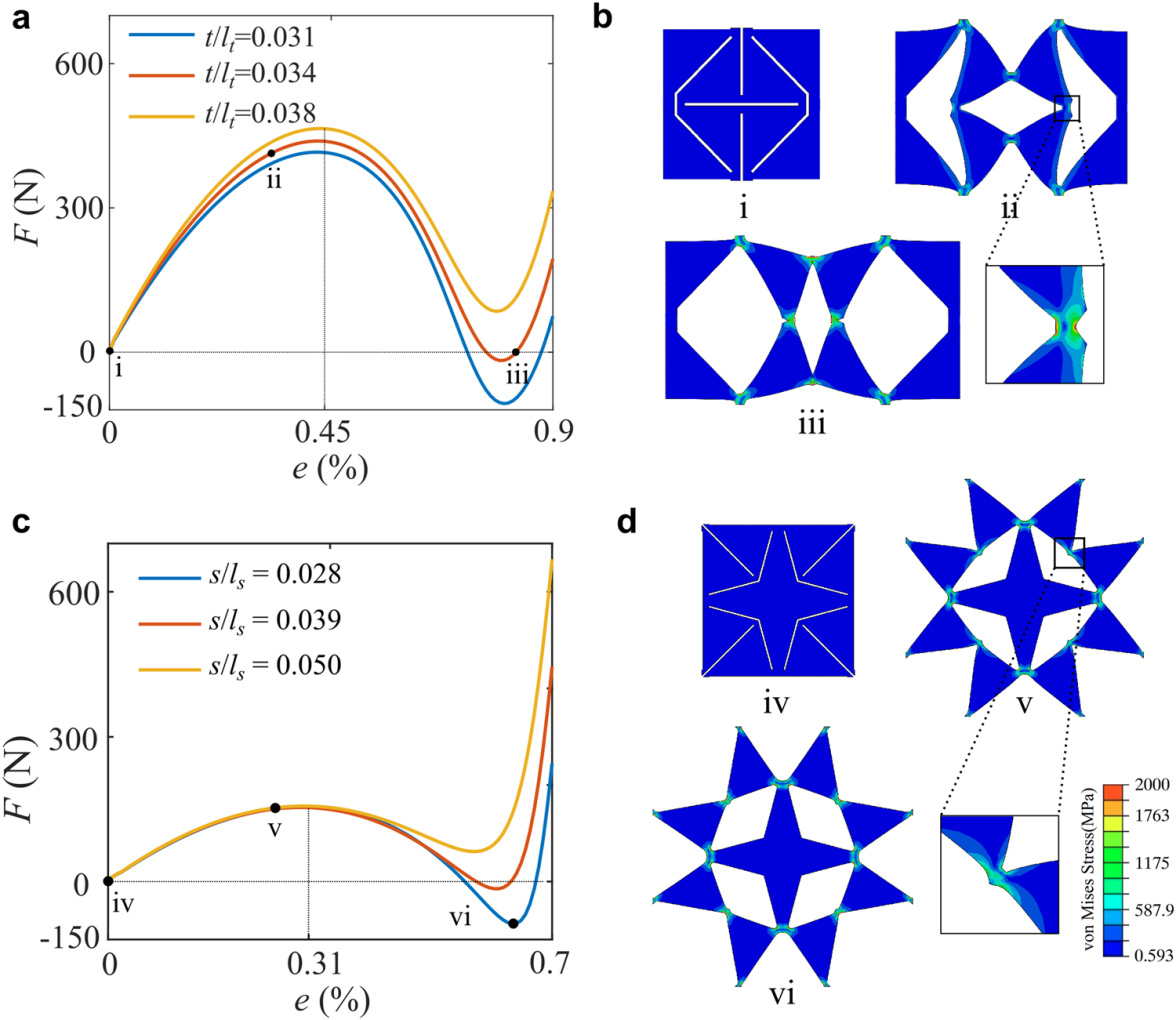
Bistability analysis. (**a**,**b**) Finite Element simulations of the triangular pattern. (**c**,**d**) The Finite Element simulations of the star pattern.

A nonlinear Finite Element (FE) analysis was performed to explore the bistability of the structure. The simulation was performed using the ABAQUS Standard Implicit Dynamics solver with moderate dissipation which improves convergence when self-contact is present. We use the neo-Hooke method for the hyperelastic feature of rubber sheets, and the geometric nonlinearities were taken into account. The models were discretized with CPS8R and CPS6. For single unit cell models, periodic boundary conditions were applied. A simplified contact law was assigned to the model with hard contact for normal behaviour. A parametric model was created using the Python scripting interface of ABAQUS to further investigate the role of different geometrical parameters on the response of the designed mechanical metamaterials. For the triangular pattern, both the length and width of the unit cell are *l*_t_ = 16 mm, its thickness is 2.3 mm, the width of the cut is 0.2 mm, and *α* = *π*/4. As discussed previously, the multi-stability of the structure is mainly contributed by the elasticity of the flexure hinges, which is highly dependent on the dimension of these hinges *t*. To validate the factors influencing the bistability, we simulated the response of a unit cell under uniaxial length variation in the horizontal direction (displacement loading) and simultaneously tracked their reaction force *F* and engineering strain *e*, defined as the length variation over the original length of the unit cell. Three cases were considered with *t*/*l*_t_ = 0.031, 0.034 and 0.038, respectively. As shown in Fig. 2a, the force-strain curve is strongly nonlinear, and the reaction force drops below zero then returns to positive values when *t*/*l*_t_ = 0.031 and 0.034. This phenomenon indicates that there are a number of stable positions that the structure could take without any stretching force. When *t*/*l*_t_ = 0.038, *F* is always above zero, indicating the intermediate stable position does not exist. Hence, tuning *t* can program the stable positions of the structure. Moreover, the larger *t* is, the higher the stretching force is required to deploy the sheet. The distribution of the von Mises stress of a unit cell with *t*/*l*_t_ = 0.034 in Fig. 2b shows that the high stress localizes mainly at the hinges and the rest of the structure has almost negligible deformation, which is in accordance with the assumption that the triangular pieces can be treated as rigid bodies kinematically. For the rubber engravable rubber prototype, *t* was chosen as *t* = 0.55 mm (*t*/*l*_t_ = 0.034).

To further optimize the bistable behaviour of both cut patterns in detail, we built a simplified elastic energy model of both structures, and this parametric analysis is provided in Supplementary Materials S2. The energy model indicates that different hinge dimensions have different influence on the bistability of the metamaterial.

The star model is analysed using the same method. Both the length and width of the unit cell are *l*_*s*_ = 14 mm and *β* = 24°. We set the hinge dimension as *s*, and let *s*/*l*_*s*_ = 0.028, 0.039 and 0.050. We simulated the response of a star unit cell under uniform horizontal and vertical displacement loading at the four corners of the unit cell and tracked their reaction force *F* and engineering strain *e*. As shown in Fig. 2c, the reaction force drops below zero then return to positive values for *s*/*l*_s_ = 0.028 and 0.039. The deployment of each pair of triangles is similar to that in the triangle pattern. *s* has a great influence on the multistability of the structure; the smaller *s* is, it’s more likely to have bistable behaviour. However, we cannot make *s* too small for the connection between pieces would become too fragile after several rounds of deployment. The minimum *s* required to have intermediate stable position is 0.5 mm (*s*/*l*_s_ = 0.036), and thus, for the laser engravable rubber prototype we chose *s* = 0.55 mm (*s*/*l*_s_ = 0.039). Figure 2d shows the distribution of the von Mises stress in star unit cell, and the stress again localizes mainly at the hinges.

The influence of the star hinge dimensions and arm angle *β* are also investigated for the structural stability in Supplementary Materials S2. Geometrically a smaller *β* enables the structure to deploy to a greater extent, which increases the geometry discrepancy requiring large snap-through force.

In Supplementary Materials S2, the tensile tests of laser cut models are also demonstrated, and they are in agreement with the numerical results.

### Tuneable dipole

#### Structure description and fabrication

A reconfigurable and tuneable half-wave dipole is presented as a potential application of the multistable kirigami structures, in particular of the triangular kirigami shown in Fig. 1a. This application is obviously inspired by classic tuneable half-wave dipole antennas with telescopic elements^33^. An advanced electronic version of the telescopic dipole concept has been recently proposed in the form of a *p-i-n* diode array structure whose elements are “activated” to achieve high conductivity by applying forward voltage between the *p*-type and *n*-type regions^34^. The antenna operating frequency is reconfigured by changing the number of *p-i-n* diode cells which are forward biased, which requires to connect bias lines to each of the antenna elements with choke inductors and decoupling capacitances. Heat generation in the diode due to the high forward bias and radiation interference with metallic elements, such as the DC-bias lines, are among the drawback of this structure. In another recent work on the realization of a frequency-tuneable half-wavelength dipole antenna, the use an array of electrically actuated liquid–metal pixels has been proposed^35^.

**Figure 3:**
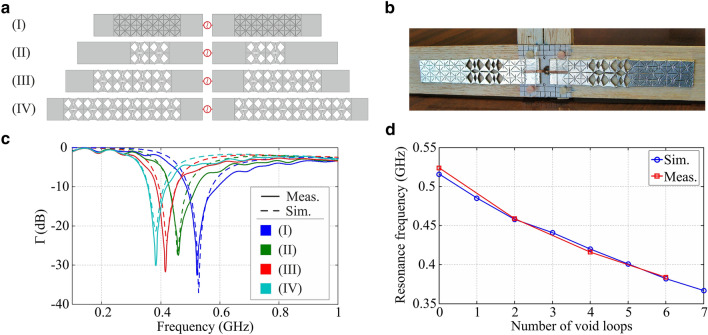
(**a**) Sketches of realized dipole model. (**b**) Sample prototype in the measurement setup. (**c**) Simulated and measured reflection coefficient of the tuneable dipole in the sample configurations of variable extension shown in subfigure (**a**). (**d**) Comparison of the simulated and measured range of tunability.

In this work the tunability of the dipole response is achieved by varying the extension of the kirigami rubber strips that covered by a thin metallized layer form the dipole arms. The dipole made by two fully closed kirigami strips is shown in Fig. 3a along with the sample configuration featuring six unit cells stretched open in each of the two rows of the kirigami pattern. In the other sample dipole configurations which have been analysed the dipole features intermediate extensions corresponding to two and four unit cells stretched open in the kirigami patterns. The kirigami strips forming the dipole arms contains a total of nine unit cells and therefore could be in principle extended to larger lengths. It is noteworthy that a similar approach could be applied to realize a frequency reconfigurable microstrip patch antenna by using the star kirigami pattern.

The proof-of-concept dipoles have been realized by simply gluing an aluminium foil onto the rubber sheet and then cutting through the foil by following the kirigami pattern imprinted in the rubber sheet with the laser. An example is shown in Fig. 3b, where it can also be seen that the dipoles are directly fed by a rigid coaxial cable with a 50 Ohm impedance.

#### Simulation and measurements results

The response of the sample dipoles have been characterised by observing their reflection coefficient Γ measured with a vector network analyser (VNA). To compare with measurements, the reflection coefficient of the dipoles has been also simulated with CST Microwave Studio (MWS), where ideal geometrical configurations, without any deformation in the kirigami pattern that real structures can exhibit when stretched, have been assumed and a short section of a 50-Ohm coaxial cable to feed the dipoles have been included in the model. The simulated and measured reflection coefficients for the dipoles in the four sample configurations are displayed in Fig. 3c. The observed frequency range shows the fundamental (lowest frequency) resonance of the dipoles. Resonance frequencies deduced from measurements are in very good agreement with the corresponding simulation data. Some oscillations in the measured reflection coefficients, which are not present in the simulated curves, might be due to noise or some residual inaccuracy in the calibration of the VNA. It can be seen that the resonance frequencies shift downwards while the kirigami strips forming the dipole arms are extended by increasing the number of open unit cells. As a result, the fundamental resonance of the dipole can be tuned from about 0.52 to 0.38 GHz, corresponding to a tuneable bandwidth of approximately 30%, with respect to the centre frequency of the tuneable range. Meanwhile the dipole fractional bandwidth only marginally shrinks. Based on simulations, the radiation pattern at the resonance (not reported as it is the standard pattern for dipole antennas) remain practically the same for all considered dipoles. Simulations of alternative dipole configurations have shown that the positions of the open unit cells along the kirigami strips forming the dipole arms have practically no effect on the dipole response, which is only affected by the number of open cells and thus the overall extension of the dipole arms.

#### Tunability analysis and discussion

The main geometrical and electrical parameters characterizing the dipole antenna at the fundamental resonance for variable number of open unit cells are summarized in Table 1. The trend of the dipole resonance frequency against the number of open unit cells in each row of the kirigami strips, resulting in the different dipole physical extensions shown in in Table 1, is illustrated in Fig. 3d, where simulation results for a few extra dipole configurations are also included. Taking into account these additional data, the dipole tuneable bandwidth reaches approximately 35% with respect to the centre frequency of the tuneable range.

**Table 1:** Geometrical and electrical parameters characterizing the dipole antennas at the fundamental resonance for variable number of void loops in the kirigami strips forming the dipole arms.

Open cell no.	Length, *L* (mm)	Resonance frequency, *f*_*r*_ (MHz)		Electrical length (*L/*λ_*r*_)	BW (MHz)		FBW (%)	
		Sim.	Meas.		Sim.	Meas.	Sim.	Meas.
0	232	520	524	0.402	80	108	15	20.6
1	248.85	485	n.a.	0.403	70	n.a.	14.4	n.a.
2	265.7	458	459	0.406	67	94	14.5	20.4
3	278.55	441	n.a.	0.41	63	n.a.	14.3	n.a.
4	295.4	420	416	0.414	59	70	14	16.8
5	312.25	401	n.a.	0.418	56	n.a.	13.9	n.a.
6	329	382	384	0.42	52	63	13.6	16.4
7	345.95	367	n.a.	0.423	48	n.a.	13.1	n.a.

Data from measurements and simulations are compared.

### Reconfigurable frequency selective surfaces

#### Concept and structure description

The periodic multi-stable kirigami patterns introduced in this work are also suitable for the realization of mechanically reconfigurable frequency selective surfaces (FSSs). The FSSs can be realized by creating an electrically conductive/metallic layer on one side of the rubber sheet onto which the kirigami pattern is cut; another option would be to form the kirigami structure with a high permittivity material. In this work we focus on the former approach. Once covered with a suitable metallization, the kirigami metasurfaces would behave as FSSs exhibiting specific transmittance and reflectance spectra in each of their stable states. The EM response of the FSSs can therefore be reconfigured through the mechanical transformation of the kirigami patterns, provided that the metallized layer can withstand the deformation process (stretching and compression) and conductor connectivity is preserved, particularly through the thin ligaments between the triangles forming the kirigami metasurfaces. We tested that both metasurfaces can be deployed in a short period repetitively (Supplementary Materials S2).

Due to the finite width of the cuts that create the kirigami patterns, when the FSSs are closed, they do not generally behave as a uniform conductive screen stopping any radiation from being transmitted into the half space beyond it, but each of them would rather exhibit a peculiar resonant response associated with the specific length, width, and orientation of the cuts. However, simple modification of the original kirigami patterns can be devised to achieve a bistate switchable *on*/*off* FSS response, as it will be shown in the following.

#### Triangular kirigami FSS

To illustrate the concept of the proposed reconfigurable kirigami FSSs, we start by considering the metasurface with the unit cell formed by small triangles, which can be stretched only horizontally (*l*_*t*_ = 12 mm). As mentioned above, this kirigami periodic pattern can be readily turned into a reconfigurable anisotropic FSS by applying a conducting layer on top of the rubber sheet used to realize the mechanically transformable pattern. While this structure can assume a variety of configurations depending on the number of columns which are deployed, as seen in the tuneable dipole antenna, for FSS application we will focus on its completely closed and fully open states.

The EM response of the FSSs formed by the metallized closed and open triangle kirigami metasurfaces have been simulated with CST MWS using a single unit cell of the kirigami pattern with doubly periodic boundary conditions. As we aimed at using standard low cost printed circuit technology to validate simulations, the conducting layer is assumed to be formed by a 0.035-mm-thick sheet of pure copper $(\sigma_{Cu} = 5.96 \times 10^{7} {\text{ S/m}})$ placed on top of an RF laminate with $\varepsilon_{r} = 4.3$ and $\tan \delta = 0.025$ (glass-reinforced epoxy, FR4).

**Figure 4:**
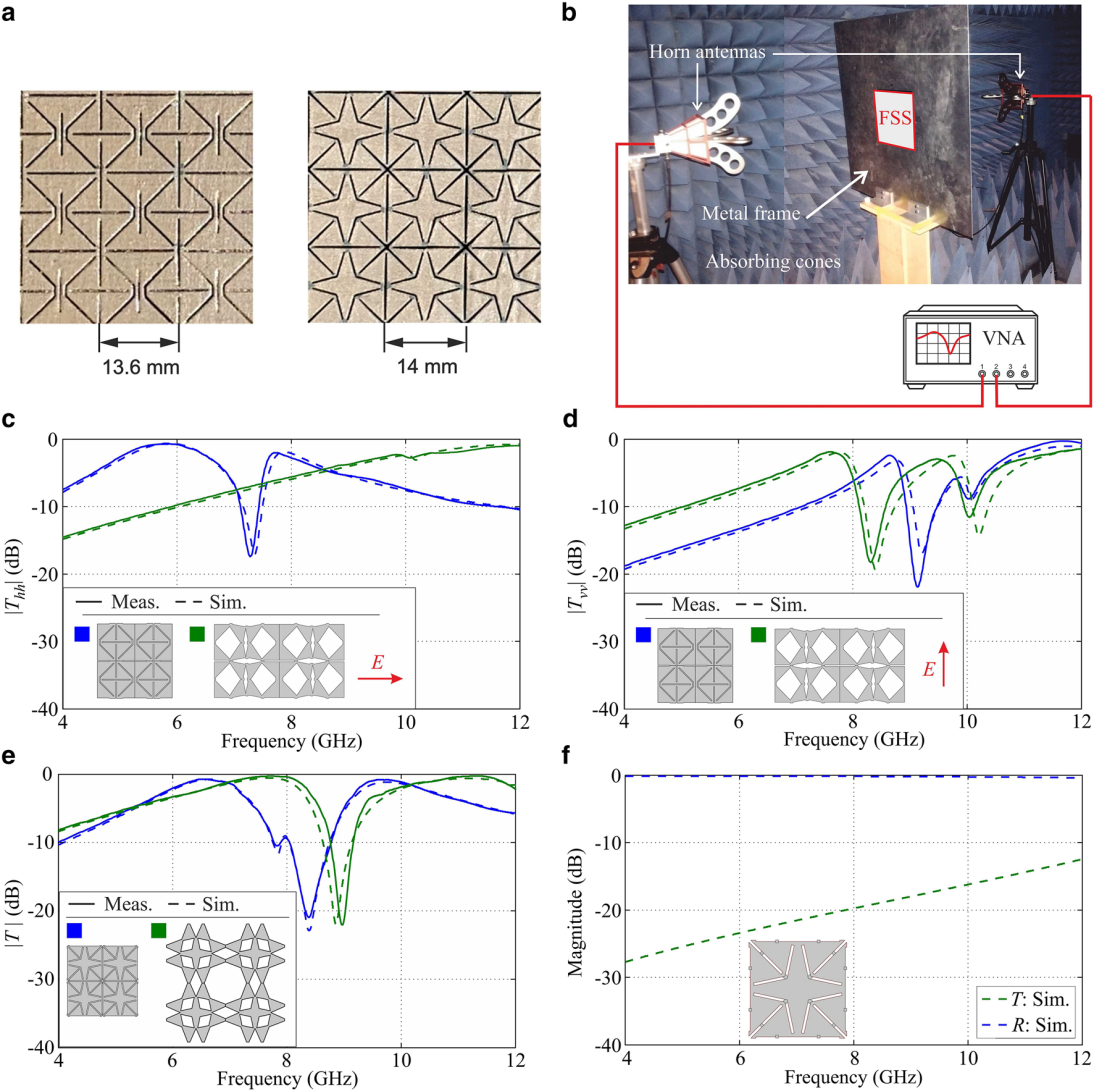
(**a**) FSS specimens. (**b**) Experiment setup. Measured and simulated transmittances of the triangle kirigami FSS in its closed and fully open configurations to a (**c**) horizontally and (**d**) vertically polarized incident wave. (**e**) Measured and simulated transmittance of the star kirigami FSS in its closed and fully open configurations. (**f**) Simulation of the switchable on–off FSS obtained by modifying the star pattern with the introduction of little metallic notches or tiny springs, as shown in the unit cell inset, which act as short circuits of the slots corresponding to the cuts defining the kirigami pattern in the closed configuration, while their presence has practically no effect when the FSS is stretched open.

As proof-of-concept, we developed rigid PCB versions of the closed and fully open states of the transformable FSS, which are shown in Fig. 4a. The fabricated FSSs were characterized by measuring their transmission properties. An actual picture of the measurement setup is shown in Fig. 4b. The specimens, all with dimensions of 20 cm × 20 cm, were fitted in a metallic frame placed between two wideband horns, connected to a vector network analyser (VNA). The diffraction by the frame window was calibrated out first by characterizing the fixture without samples. Parasitic reflections were undetectable in these tests. The measured transmittance, for both horizontal and vertical polarizations, are superimposed with the corresponding simulation results in Fig. 4c,d, respectively. It can be seen that the agreement between measurements and simulations is good, especially around the FSS fundamental resonance. It is interesting to note that when this type of kirigami FSS is in the closed state it is substantially opaque to a vertically polarized incident field at the frequencies at which the open configuration exhibits its fundamental passband resonance for the same polarization, while the opposite happens for the vertical polarization. In particular, from the graph in Fig. 4c it can be seen that the FSS in its closed state exhibits the fundamental resonance for a horizontal incident polarization, with full transmission, at about 6 GHz, whereas for the vertical polarization it behaves substantially as a perfect reflector at the same frequency. On the other hand, when the FSS is deployed into its fully open state, both polarizations are reflected, because the first resonance for this configuration, for a vertically polarized incident wave, occurs at higher frequencies and transmission is negligible in the low frequency region. In other words, the response of this triangular FSS at low frequency can be mechanically switched from fully reflecting to that of a transmitting polarizer.

#### Star kirigami FSS

The concept of the proposed reconfigurable kirigami FSSs is further illustrated by the example of the FSSs made by adding a conducting layer on top of the kirigami metasurface with the unit cell formed by a four-pointed star connected to eight small triangles. As shown in “Star pattern” section, when this pattern is tessellated in the plane, we focus specifically on the responses of the isotropic open and close states. As for the triangle kirigami FSS, for validating the reconfigurability of the star FSS, we produced static PCB versions of these two stable star kirigami configurations. The printed circuit FSS prototypes displayed in Fig. 4a have been manufactured by using standard 1–6-mm-thick FR4 laminates with 0.035-mm-thick copper cladding.

The simulation of these FSSs was again conducted by resorting to doubly periodic boundary conditions in CST MWS to reduce the domain of analysis to a single unit cell of the kirigami pattern.

Since the closed and fully open star patterns are isotropic, only the transmittance at one incident polarization needs to be examined. Similarly, the PCB specimens have been characterized by measuring their transmission coefficient to a single incident polarization. The measurements were conducted in an anechoic chamber with the same setup of Fig. 4b used for the triangle kirigami FSS.

The measured transmission coefficients are shown in Fig. 4e superimposed with the corresponding simulation results. As apparent, there is good agreement between measurements and simulations, both around the fundamental resonance and at higher frequencies. When the kirigami unit cells are closed, the FSS exhibits its fundamental passband resonance at about 6.7 GHz, whereas for the open pattern the resonance occurs at 7.8 GHz. In other words, in principle, a 15% shift in frequency of the FSS fundamental passband response can be obtained by mechanically transforming the kirigami structure, although the change of state does not result in a substantial attenuation of transmission at the frequencies of each FSS passband.

Overall, these measurements of actual finite size FSS samples confirm predictions based on the simulation of the corresponding ideal infinite periodic structures, and prove that transformable kirigami patterns hold the potential to enable a whole new class of reconfigurable FSSs not relying on any electronic components for their operation. However, an actual implementation of this concept would require that the metallization is applied directly on top of the laserable rubber sheets from which the kirigami are fabricated by using a laser cutter and that the deformability of the structure is preserved in the process.

As shown in Fig. 4e, when the star kirigami FSS is closed, it does not behave as a uniform conducting screen stopping radiation from being transmitted into the half space beyond it, but it exhibits a resonant response associated with the configuration of the slots in the metal layer corresponding to the cuts that create the kirigami pattern.

A simple modification of the original start kirigami pattern can be made to achieve a bistate switchable *on*/*off* FSS response. The idea is to introduce a regular distribution of small metallic notches along the edges of the kirigami pattern, as shown in the modified star unit cell displayed in the inset of Fig. 4f. In the closed state, these notches short-circuit the slots in the metal screen corresponding to the cuts of the kirigami pattern, while the open pattern is negligibly modified by their presence. The simulated transmission and reflection coefficients of the modified star FSS in the closed configuration are shown in Fig. 4f. As apparent, the effect of the short-circuits is to shift upwards in frequency the fundamental resonance associated with the periodic slot pattern in the closed state, as the actual lengths of the various slots is less than one half of the corresponding ones in the original pattern. This way, the level of transmittance in the frequency range of interest is considerably reduced. This approach could indeed be used to realize a bi-state mechanically switchable FSS, with the FSS being practically opaque to the incident field in the closed state at the frequencies where the open pattern exhibits its passband resonance.

## Discussion and conclusions

A new class of electro-mechanical metamaterials inspired by transformable kirigami patterns has been developed for multiple electromagnetic applications. The topologies of the structure can be dynamically tuned and locked into several different configurations due to its multi-stability. For each stable configuration, the unit cell of the metamaterials exhibits different resonant properties, which we showed can be exploited for applications such as tuneable dipoles and FSSs. Both these types of structures require that an electrically continuous conductive layer is applied on one side of the rubber sheet onto which the kirigami pattern is cut, and that the electrical continuity between the cells is preserved while the structure undergo mechanical deformation to reconfigure its EM response. This is very challenging at the thin ligaments that act as flexure hinges between the elements forming the kirigami patterns, which are bent and compressed during the transformation.

For our proof-of-concepts, we developed static PCB versions of the transformable FSSs in their different configurations, while we used flexible aluminium foil attached to the rubber sheet for the tuneable dipole samples, which however tends to crack after a few deformations. In practice, for the operation of the proposed kirigami-inspired EM structures, a stretchable conducting surface capable of withstanding repeated mechanical elongation and compressions would be required^36^. In recent years, there have been significant progress in the development of elastic conductors, which are essential components for large-area stretchable sensor and actuator networks for healthcare, wearables and robotics. One of the common approaches to realize flexible conductors is to mix elastomers with metal nanoparticles. Many novel nanomaterials have been studied to achieve flexibility and good electrical performance. Silver-based nanomaterials, including silver nanoparticles, nanowires, and nanoflakes, have been found to be promising candidates for use in stretchable conductors^37–39^. Similarly, highly stretchable conductors made of gold nanostructures have been also reported^40,41^. A more detailed summary of the significant progress in the development of stretchable conductors can be found in^42^.

Another approach to realize stretchable electronics is to use rigid active device islands and stretchable interconnects, such as in^43,44^. Similarly, in our kirigami structures we could introduce a periodic pattern of disconnected rigid metal patches on the top of the rubber substrate, leaving small portions of the rubber surface around the flexure hinges bare, i.e. without any metallic covering, and then connect the isolated metal patches by either simply soldering wires between them or applying other types of flexible interconnects. The technological aspects of the realization of the proposed EM reconfigurable kirigami devices are beyond the scope of this work and will be addressed in a future publication.

The focus in this paper has been to show, by both parametric analysis and energy model construction, that we can program the stable configurations of the kirigami patterns developed. The relation between pattern parameters and the electromagnetic performance has been investigated and tested. By utilizing kinematic based design tools, we can create various morphing structures with large deformation range, and there is a large design space that remains to be explored. Correspondingly, the shape variation of electro-mechanical metamaterials provides a broad tuning range for the electric properties and EM responses of tuneable antennas, filters and other components, as required by the next generations of wireless communication systems, remote sensing, and wearable electronics for biomedical applications.

## Supplementary Information

Supplementary Information 1.

Supplementary Video 1.

Supplementary Video 2.

## Data Availability

All data is available in the main text or the supplementary materials.
