# Supplementary material for: A new class of transformable kirigami metamaterials for reconfigurable electromagnetic systems
Source: Sci Rep. 2023 Jan 21;13:1219. doi: 10.1038/s41598-022-27291-8 (PMC9867698; doi:10.1038/s41598-022-27291-8)
Supplement: Supplementary file 1 — Supplementary Information 1. [file 41598_2022_27291_MOESM1_ESM.docx]

**Supplementary Information**

**A New Class of Transformable Kirigami Metamaterials**

**for Reconfigurable Electromagnetic Systems**

**Supplementary Material S1**

**Sample Fabrication**

The samples are fabricated by perforating the designed patterns into low odour laserable rubber sheet (A4 size, 2.3 mm thick, shown in Fig. S1(a)) using a laser cutter. The cut width is around 0.2-0.4 mm. We tested that the sufficient thickness for the hinge to be fabricated and bearable under repeat loading is 0.5 mm. For the triangle model, the size of the unit cell is 12×12 mm^2^, which brings the resonance of the metasurface around 10-12GHz (the measurement range is about 8-14 GHz). The angle *α* is π/4. The ligament width for the hinges is *t_i_* = 0.8 mm (*i* = 2-6). To achieve strong bistability, we make *t*_1_ = 6mm, meaning there is no cut between two neighbouring units in one row. The metamaterial is composed of 17 units in each direction, and the total dimension of the sample is 204×228mm. For the star pattern, the size of the unit cell is 14×14mm and the star angle *β* is 24°. To make the structure have robust bistability, we design the hinge width to be *s_i_* = 0.7mm (*i* = 1,2,3). The metamaterial is composed of 15 units in each direction with a dimension of 210×210mm.

To coat the sample with metal, we pre-processed the rubber sheets before laser cutting. First, we clean and polish the rubber surface thoroughly and brush metal paint (Copper conductive paint, Caswell) on it. The paint is coated three times to make sure the conductivity on the rubber sheet is good. Then the coated sheet is cut with the designed pattern. After this, the cut sheet is cleaned and painted again for two times to keep the conductivity at thin ligaments. Finally, we tested the surface resistivity of the copper paint coating by using the four-probe method and a source meter (Keithley 2400). The conductivity of the applied copper paint was measured to be a factor 10^4^ smaller than that of pure copper. If the sample has no defect, it is ready to be measured as a Frequency Selective Surface. As a reference, transmittance simulated by assuming that the metallized layer is made of pure copper is included in Fig. S1(e). The whole fabrication process and the fabricated samples are shown in Figure S1.


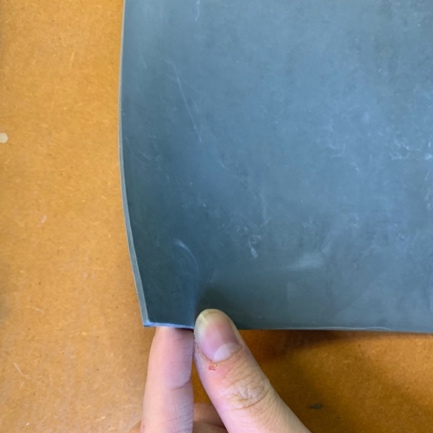

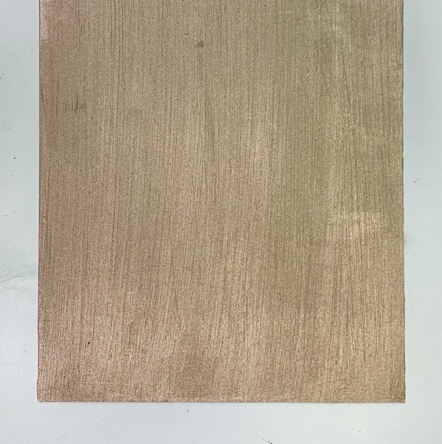

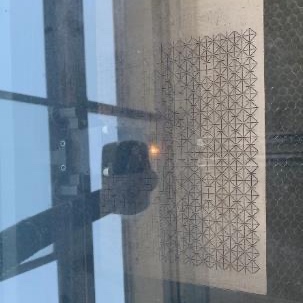


(a) The laserable rubber sheet (b) Sheet coated by metal paint (c) Engraving the rubber sheet


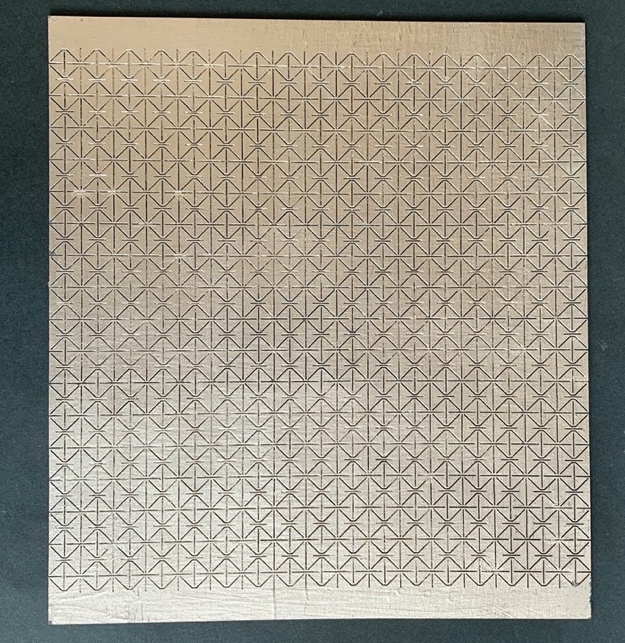

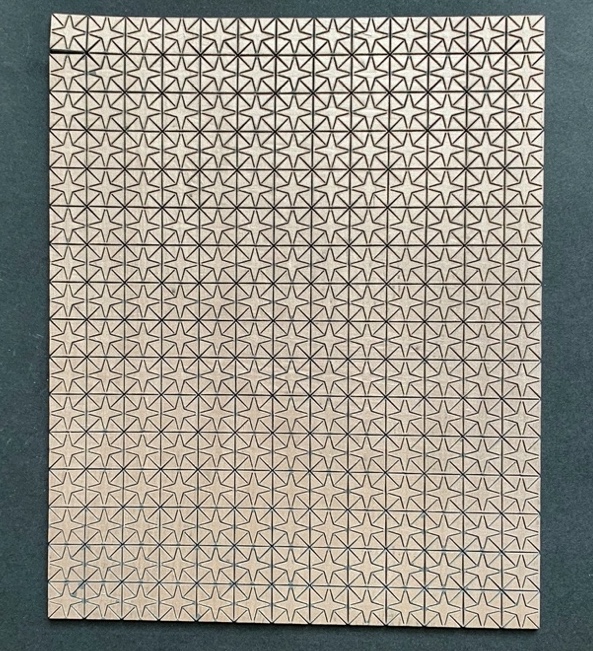


(d) The fabricated samples of the triangle and star models.


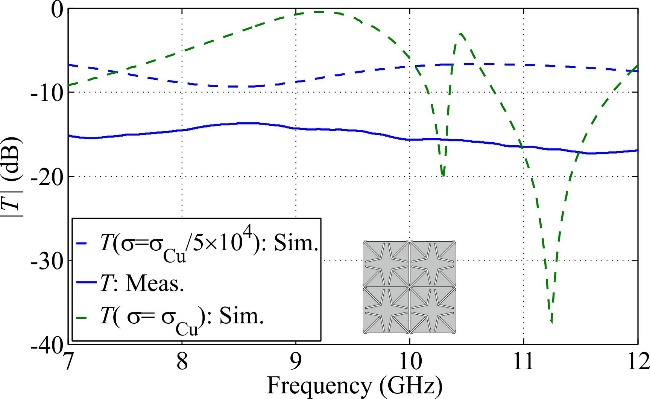

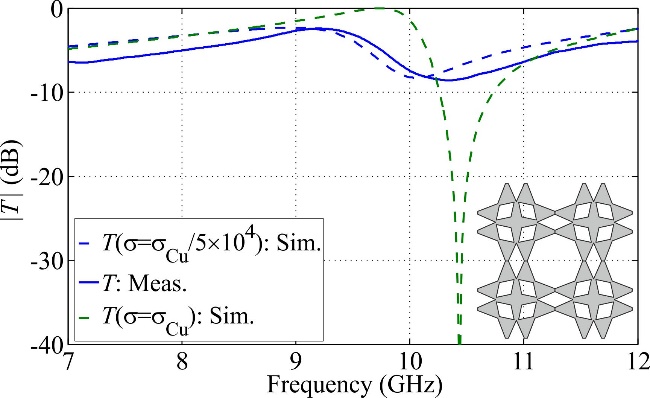


(e) Measured and simulated transmittance of the star kirigami FSSs made by coating the rubber sheets with copper paint at normal plane wave incidence in its open and closed states.

**Figure S1 Fabrication process of the Frequency selective surface.**

**Supplementary Material S2**

**Structural transformation**

The transformation of the metamaterial can be modelled by planar linkage mechanisms. If we assume the triangles have an ideal vertex-to-vertex connection, the deformation of the structure can be regarded as a rigid body motion, of which the mobility is given by

$m=3(n-1-j)+\sum_{i=1}^{j} f_{i}=3(8-1-10)+10=1$ (S1)

The motion can be described in three steps, as shown in Figure S2:

1) When applying transversal load on the close structure, triangles 1 & 2, 3 & 4, 5 & 6 and 7 & 8 first stick together as squares and move as integrated bodies. The neighbouring squares rotate around the connection vertices with respect to each other and form a planar 4*R* linkage.

2) When the hypotenuses of the triangles in the top and bottom squares become collinear, the structure can choose from two motion paths: either continue the rotation as one part or separate the triangles and form new parallelogram voids within. Because we apply transverse load on the structure, it tends to open up further and separate the triangles. Therefore, two new 4*R* linkages are generated within triangles 1-4 and 5-8. Triangles 1,4,5,8 change their rotation direction from clockwise to anti-clockwise, or vice versa.

3) Finally, the structure reaches the fully open state where its transversal dimension is maximized. The three parallelogram voids morph into two square voids, and triangles 1, 4 contact with 8, 5.


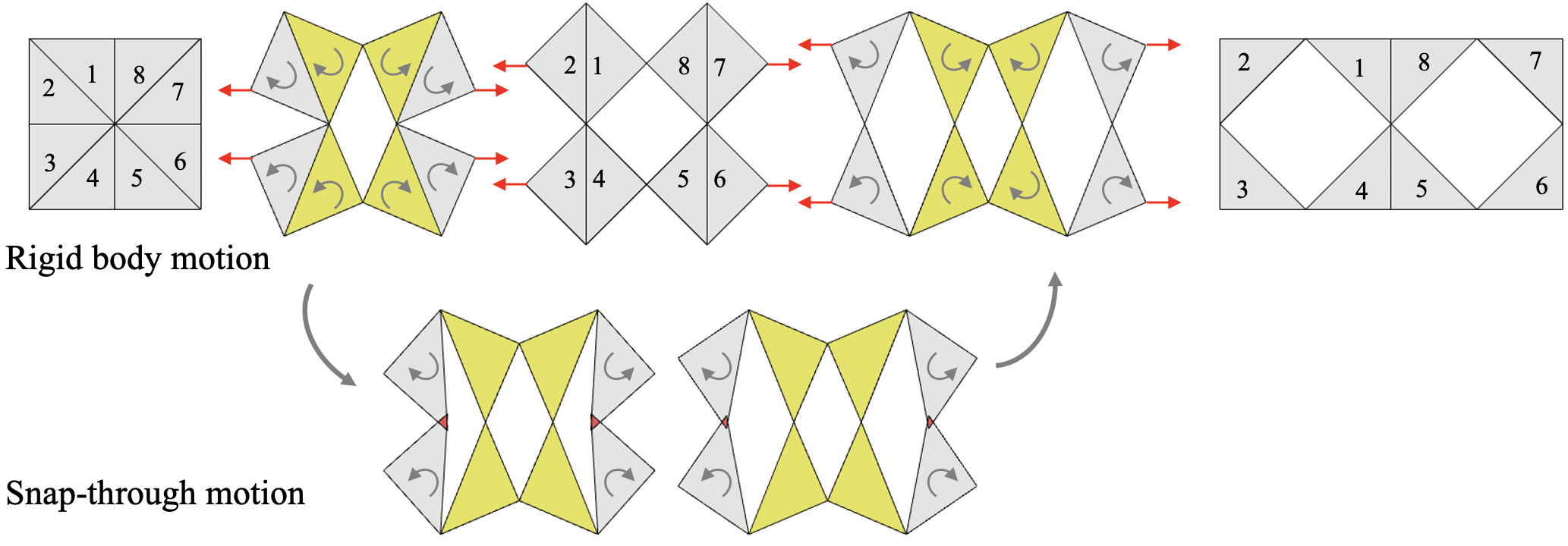


**Figure S2 Kinematic analysis of the structure**

**Simplified energy model**

To further optimize the bistable behaviour of both cut pattern, we build a simplified elastic energy model of the structure. The total potential energy *Π*, is the sum of the elastic strain energy *U* stored in the deformed body and the potential energy of the applied load *V* associated with the applied forces. The system is in equilibrium if an infinitesimal change of the position does not change the total potential energy:

$\delta\Pi=\delta(U+V)=0$ (S2)

To be able to free stand at the stable states, the potential energy of the applied load at the stable state is zero. Therefore, the derivative of strain energy *U* is also zero to make the system in equilibrium. This indicates that the stable states happen when the strain energy reaches local minima. In the triangle model, the elastic strain energy U is mainly consisted of the energy in the bending ligaments by pure moment

$U=\int_{0}^{L} \frac{M^{2}}{2EI}dx=\frac{EI}{2L}d\theta^{2}$ (S3)

Where the area moment of inertia *I* can be derived as

$I=\frac{bt^{3}}{12}$ (S4)

For each unit, the strain energy of the bending hinges can be calculated as

$U_{B}=\sum_{i}^{6} \frac{EI_{i}}{2a_{i}}d\theta_{i}^{2}=\sum_{i}^{6} \frac{1}{2}k_{i}d\theta_{i}^{2}$ (S5)

$k_{i}=\frac{Eb_{i}t_{i}^{3}}{12a_{i}}$ (S6)

*k_i_* can be seen as the equivalent stiffness of the flexure hinge, and it is determined by the geometry of the hinge according to Eq. (S6). As shown in Fig.S3(a), to mimic the structure in Fig.S2, we make a cut between the adjacent unit cells, creating a new hinge with a thickness of *t*_1_. If the value of *t*_1_ is very small, the behaviour of the triangle model is similar to the planar linkage mechanism. On the other hand, if we increase the value of *t*_1_ until *t*_1_ = *l_t_* /2, this new cut is eliminated and the behaviour of the unit cell is equivalent to the one shown in main text. In Figure S3, *b_i_* denotes to the thickness of the sheet material, *t_i_* is the width of the hinge *i*, *a_i_* is the length of the hinge. The length of hinge *a_i_* is hard to define; here we use an approximate value measured from real sample behaviour. *E* is the modulus of elasticity. The dimension of the unit is *l* and one acute angle of the right triangle is *α.* Here we use isosceles right triangles so *α* = π/4.


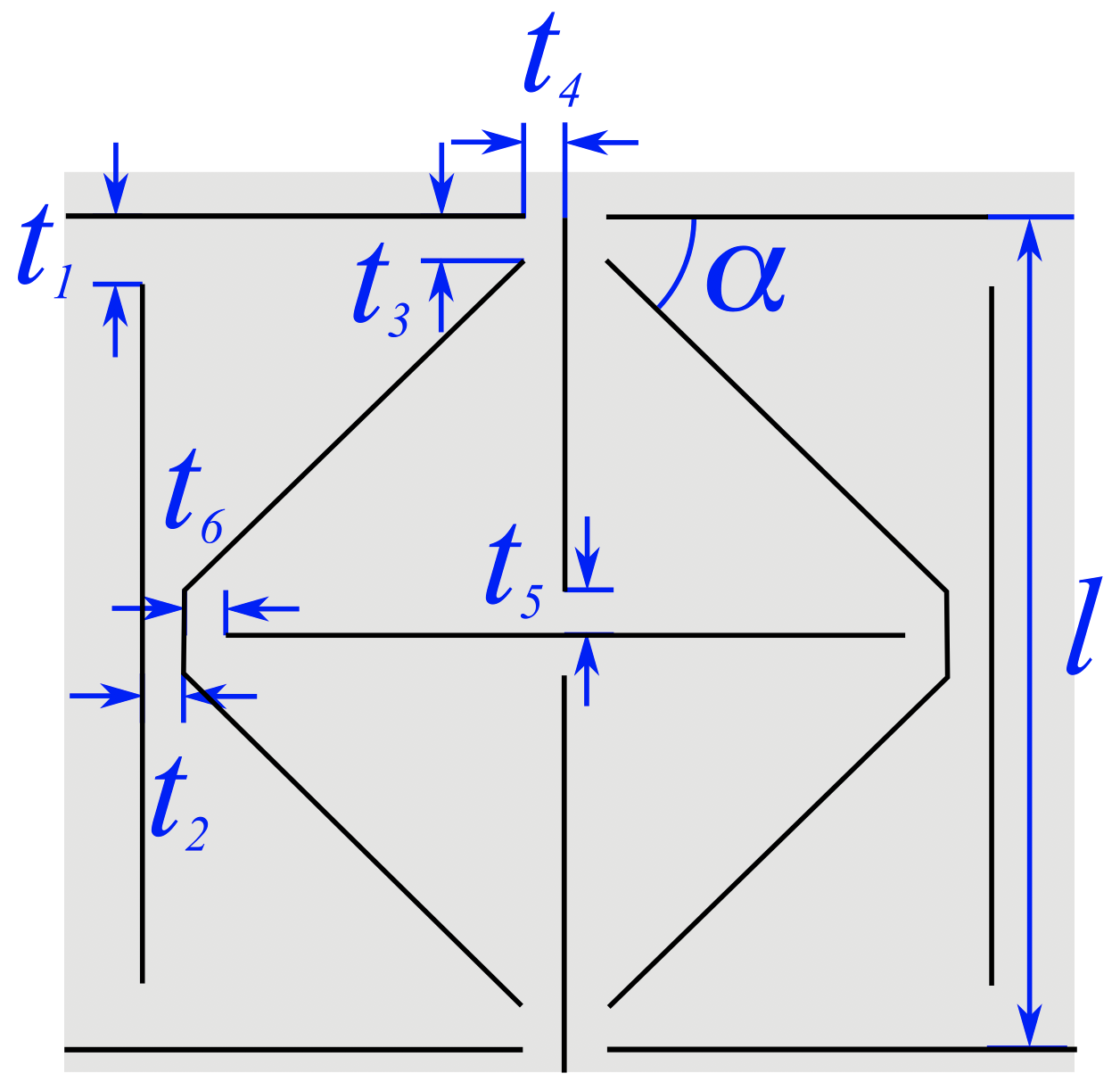

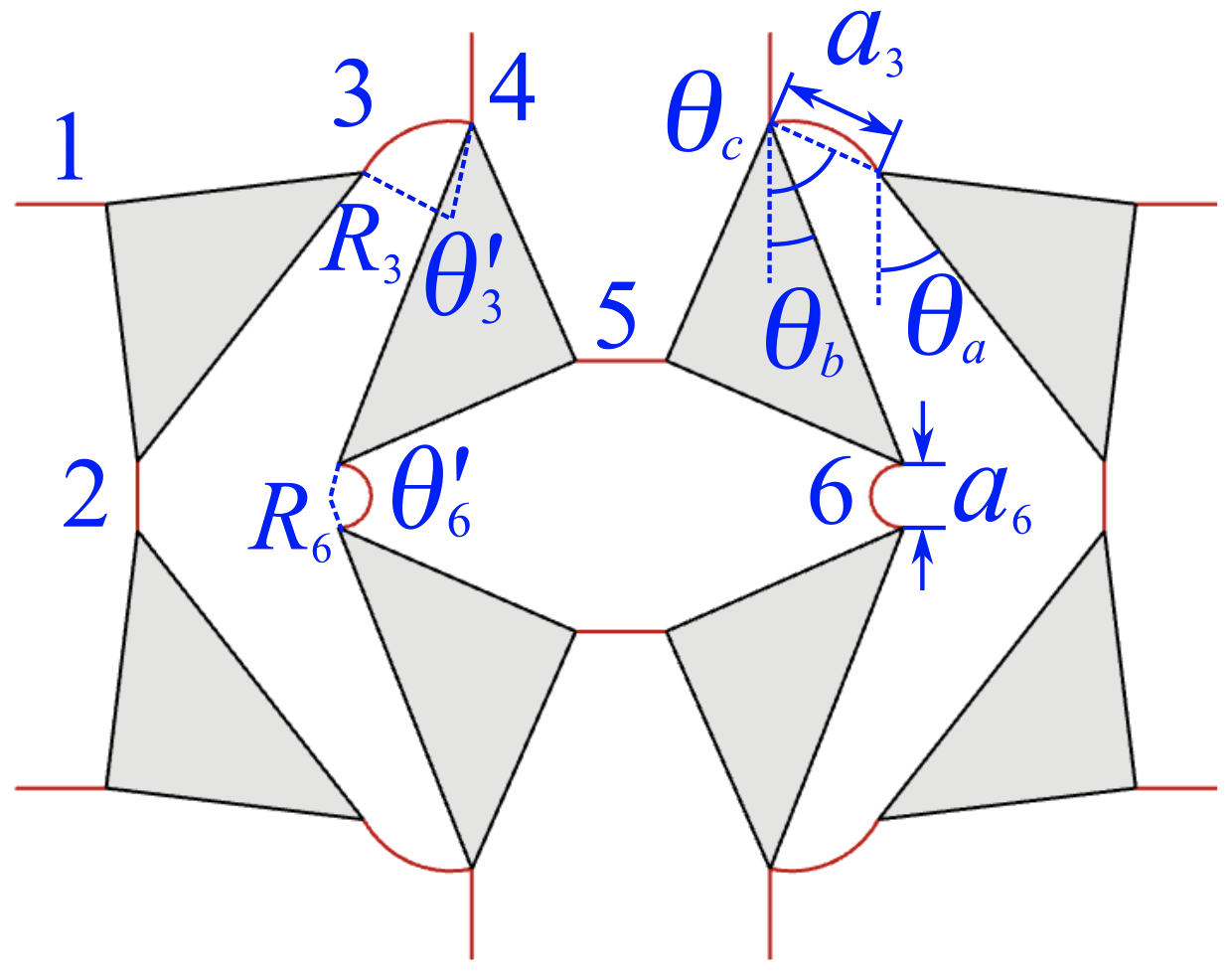


Figure S3 The parameters of triangle model unit cell.

We assume the bending arc of each flexure hinge is tangent to the adjacent triangle edges. Therefore, the bending angle of the hinge is equal to the intersection angle of two adjacent triangle edges on the same side. Especially, hinge 3 and 6 are compressed severely during the snap through, so they tend to have bigger curvatures. Their bending angle is given by adding the intersection angle and a local rotational angle $\theta'_{i}$.

$R_{i}\theta_{i}^{'}=a_{i}^{0},i=3,6$ (S7)

$2R_{i}\sin\frac{\theta_{i}^{'}}{2}=a_{i}$ (S8)

Therefore

$2\frac{a_{i}^{0}}{\theta_{i}^{'}}\sin\frac{\theta_{i}^{'}}{2}=a_{i}$ (S9)

Here, $a_{i}^{0}$is the original length of the thin ligament and *a*_i_ is the distance at the vertex between two triangles connected by hinge *i*. *R_i_* is the radius of the local arc corresponding to$\theta'_{i}$.

The void inside the hinge 2,3,6 needs to fulfil the physical constraint:

$\frac{a_{2}}{2}+\sqrt{2}l\cos\theta_{a}+a_{3}\cos\theta_{c}=\sqrt{2}l\cos\theta_{b}+\frac{a_{6}}{2},\theta_{c}\in(0,\frac{\pi}{2})$ (S10)

According to Figure S3, we can list the bending angle of each hinge:

$d\theta_{1}=d\theta_{2}=d\theta_{4}=2(\theta_{a}-\alpha)$ (S11)

$d\theta_{5}=2(\theta_{b}-\alpha)$ (S12)

$d\theta_{3}=\theta_{a}-\theta_{b}+\theta_{3}^{'}$ (S13)

$d\theta_{6}=2(\theta_{b}-\alpha)+\theta_{6}^{'}$ (S14)

$\theta_{a}\in(0,\alpha),\theta_{b}\in(-\alpha,\alpha)$ (S15)

Sum up the bending energy of each hinge we have the following equation:

$U=\sum_{i}^{6} n_{i}\frac{1}{2}k_{i}d\theta_{i}^{2}=2\times\frac{1}{2}(k_{2}d\theta_{1}^{2}+k_{5}d\theta_{2}^{2}+k_{6}d\theta_{6}^{2})+4\times\frac{1}{2}k_{3}d\theta_{3}^{2}+\frac{4}{2}\times\frac{1}{2}(k_{1}d\theta_{4}^{2}+k_{4}d\theta_{5}^{2})$ (S16)

*n_i_* is the number of identical hinges in a single unit. By scanning the whole parameter space of$\theta_{a},\theta_{b},\theta'_{3},\theta'_{6}$, we can plot the minimum energy of each corresponding *θ_b_* in Figure S4.

During the deformation, *θ_a_* will first decrease from *α* to a certain angle, then recover to *α* in the end; *θ_b_* will decrease all along until the limit. Therefore, the energy in hinge 1,2 and 4 will increase in the beginning to reach local maxima and decrease afterwards, while the energy in other hinges will keep increasing during the motion. The energy variation in hinge 1,2,4 raises the bistability, so we can tune the property by changing the stiffness of these hinges. According to Eq. (S5)-(S6), *k_i_* can be seen as the equivalent stiffness of the flexure hinge. For hinge 1,4,5 the hinge length can be seen as the cut width *a_i_* = *t_c_* ≈ 0.3mm, *i* = 1,4,5; for hinge 2,3,6, the length is much bigger than cut width, which is approximately *a*_2_ = 5*t_c_*, *a*_3_ = *a*_6_ = 3*t_c_*. The dimension of the unit cell is *l* = 12mm to ensure that the resonance frequency of the metasurface is within our measurement range. The maximum length for *t*_1_ and *t*_4_ is 6mm, which is half the size of a unit cell. Under such cases, there will be not cut between the adjacent unit cells. When increasing *t*_2_, we keep *α* unchanged. This influences the dimension of the structure and result in a wider unit. There is no maximum length for *t*_2_. The limits of hinge widths are summarized

$\begin{matrix} t_{i}=0.8\text{mm}(i=3,5,6), \\ t_{c}\approx0.3\text{mm}, \\ t_{1},t_{4}\in[0.5\text{mm},6\text{mm}], \end{matrix}$ (S17)

$k_{i}\propto\frac{t_{i}^{3}}{a_{i}}.$ (S18)

It can be noticed that the width of the hinge largely influences the hinge stiffness. Therefore, we use the width of hinge 1,2 and 4 to tune the elastic energy. The initial setting for other hinge widths is *t_i_* = 0.8mm. Figure S4 plots the influence of these parameters on the strain energy.

Figure S4(a) shows that the width of hinge 1 influences the strain energy the most. When *t*_1_ reaches the maximum, the cuts at the two sides of the unit cell disappear. This design directly constrains the deformation of the four triangles at the corner and make *θ_a_* a constant value of π/4. Therefore, the bistability of the pattern reaches the extremum. Hinge 4 has the same effect as hinge 1(Figure S4(c)).


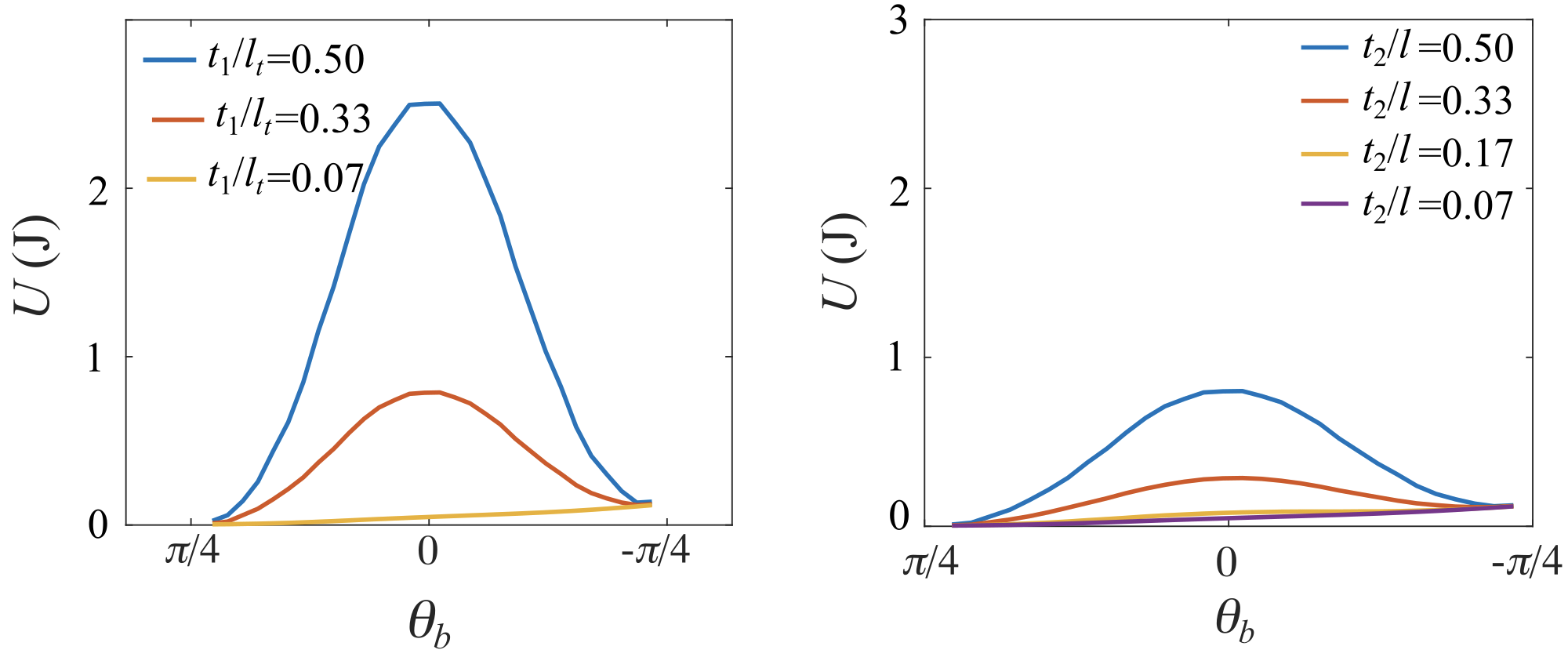


(a) *t*_1_ (b) *t*_2_


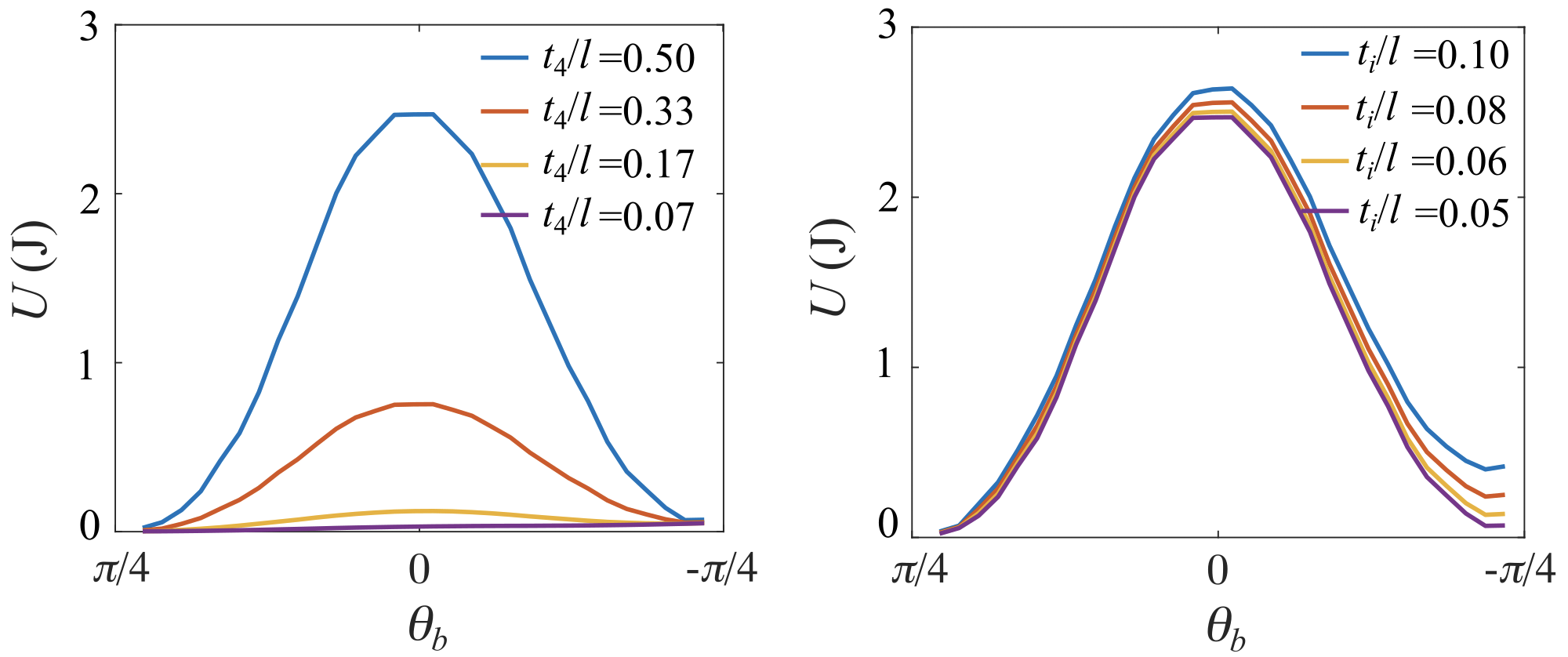


(c) *t*_4_ (d) *t*_i_ (*i*=3,5,6)

Figure S4 The elastic energy as a function of the rotational angle *θ_b_* with different hinge parameters. The initial values of these parameters are: *t_i_* =0.8 (*i*=1,2,3,4,5,6).

Hinge 2 can tune the stability as well, but not as effective as hinge 1 and 4 works. Although the stiffness of hinge 2 is largely increased, the geometry discrepancy is not very obvious and the triangles are free to transform. Thus, we did not choose this parameter to tune the bistability. The evolution of strain energy versus *t_i_* shows that the thickness of other hinges has limited influence on the bistability. From another hand, *t_i_* tunes the minimum energy of the second stable state. The thicker the hinges are designed, the harder the structure can stay stably at the open state. This does not indicate a thinner hinge will give better bistable performance because they are more likely to break after repetitive deployment. We found that when *t_i_* = 0.8mm, the pattern achieves a good balance between the stability and strength of the thin ligament.

For the star pattern, the elastic strain energy *U*, consisted of the energy in the bending ligaments, can also be calculated using Eq. (S5). It should be noted that hinge 2 in Figure S5 undergoes severe deformation during the snap-through. We calculate the bending angle of hinge 2 using the method adopted for hinge 3 and 6 in the triangle structure.

$2\frac{a_{2}^{0}}{\theta_{2}^{'}}\sin\frac{\theta_{2}^{'}}{2}=a_{2}$ (S19)


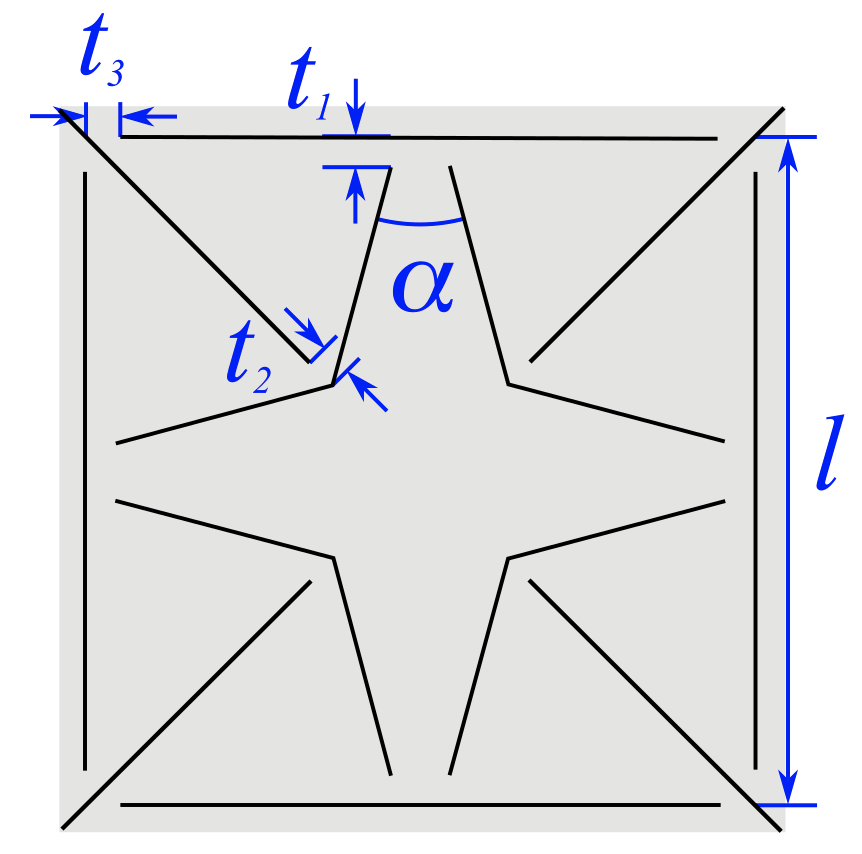

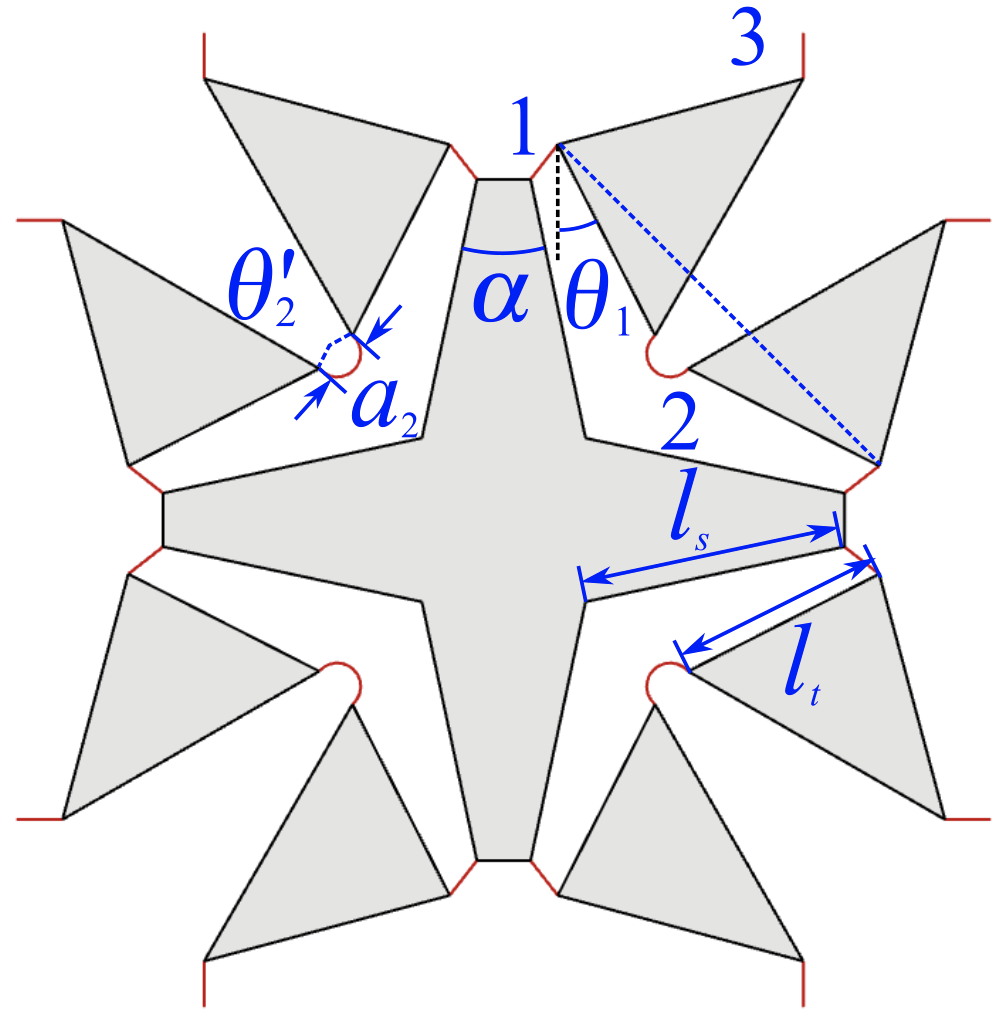


Figure S5 The parameters of star model unit cell.

The bending angle $\theta'_{2}$ is related to the distance *a*_2_ between two neighbouring triangles. $a_{2}^{0}$is the original length of hinge 2. Assuming the edge length of the star arm is *l_s_*, the length of the triangle edge which is adjacent to the star is *l_t_*, the star pattern should fulfil the following relation during transformation:

$l_{s}=\frac{\frac{l}{2\sqrt{2}}}{\cos(\frac{\pi}{4}-\frac{\alpha}{2})}$ (S20)

$l_{t}=l_{s}-\frac{\frac{a_{2}}{2}}{\cos(\frac{\pi}{4}-\frac{\alpha}{2})}$ (S21)

$a_{2}=2(\frac{l}{2\sqrt{2}}-l_{t}\cos\left|\theta_{1}-\frac{\pi}{4}+\frac{\alpha}{2}\right|)$ (S22)

*α* denotes to the angle of the star arm, *l* denotes to the dimension of the unit, and *θ*_1_ refers to the rotating angle of hinge 1. We can list the bending angle of each hinge in Figure S5:

$d\theta_{3}=2(d\theta_{1})$ (S23)

$d\theta_{2}=2(d\theta_{1})+\theta_{2}^{'}$ (S24)

$d\theta_{3}=2(d\theta_{1})$ (S25)

$\theta_{1}\in(0,\frac{\pi}{2}-\alpha)$ (S26)

Sum up the bending energy of each hinge we have the following equation:

$U=\sum_{i}^{6} n_{i}\frac{1}{2}k_{i}d\theta_{i}^{2}=8\times\frac{1}{2}k_{1}d\theta_{1}^{2}+4\times\frac{1}{2}k_{2}d\theta_{2}^{2}+\frac{8}{2}\times\frac{1}{2}k_{3}d\theta_{3}^{2}$ (S27)

*n_i_* is the number of the identical *i* hinges in a single unit. By scanning the parameter space of *θ*_1_, we can plot the minimum energy of the structure during transformation.

Among the three hinges of star model unit cell, hinge 2 has the biggest deformation and contributes the most to the bistability behaviour. When *θ*_1_ equals to *α*/2 or π/2-*α*/2, the length of *a*_2_ will return to its original length *a*, which means the deformation of hinge 2 reaches the smallest, leading to the reduction of elastic energy. Again, we can tune the strain energy landscape by changing the stiffness of the hinges through varying their width. Figure S6 shows the influence of *t*_1_, *t*_2_ and star angle *α*.

As expected, *t*_2_ determines the bistability of the pattern, while *t*_1_ has rather small influence in the performance. *t*_3_ have similar performance as *t*_1_, so we did not plot the figure here. It is worth noting that the increase of *t*_2_ will raise the overall strain energy of the open stable state. If the energy is too high, the structure will tend to snap back to the original close configuration. Hence, the pattern does not become more bistable with the increase of *t*_2_ after certain value. We found that when *t*_2_ = 0.7mm, the pattern can free stand at the stable states.


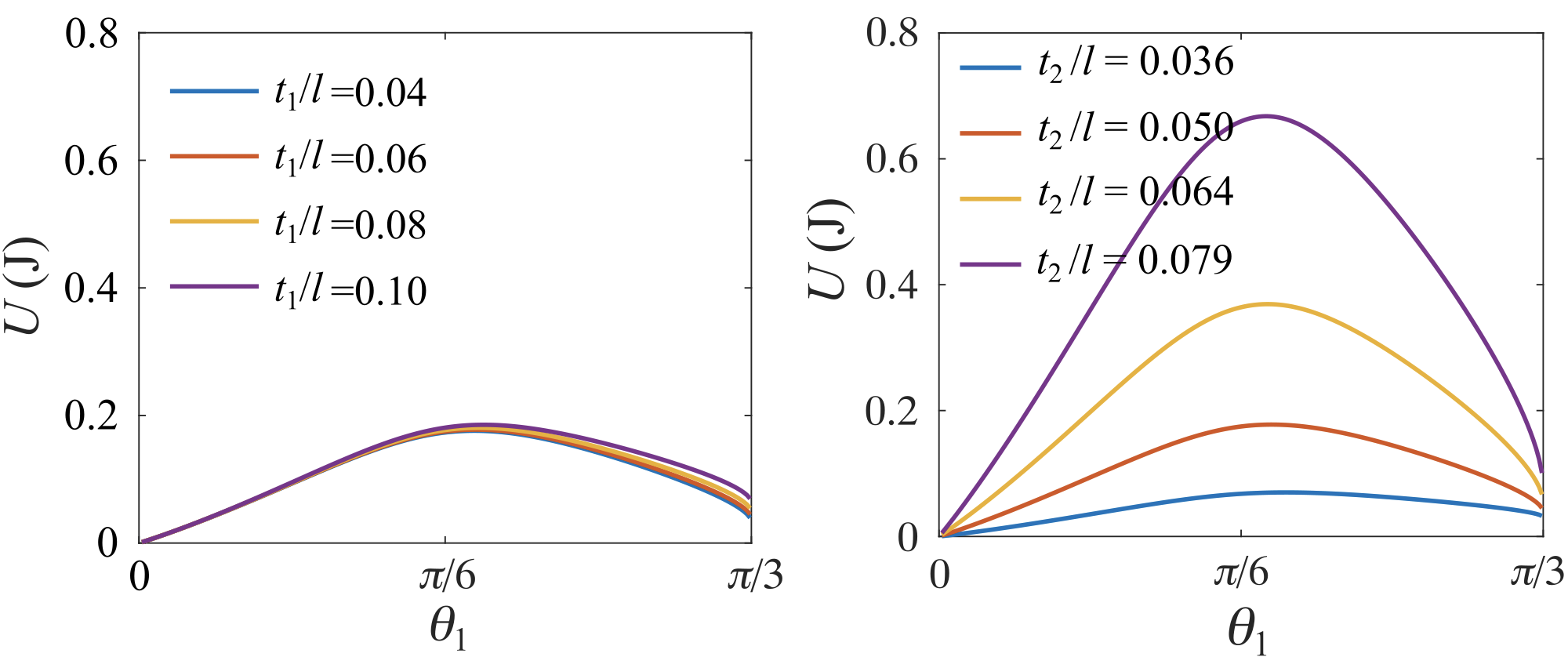


(a) *t*_1_ (b) *t*_2_


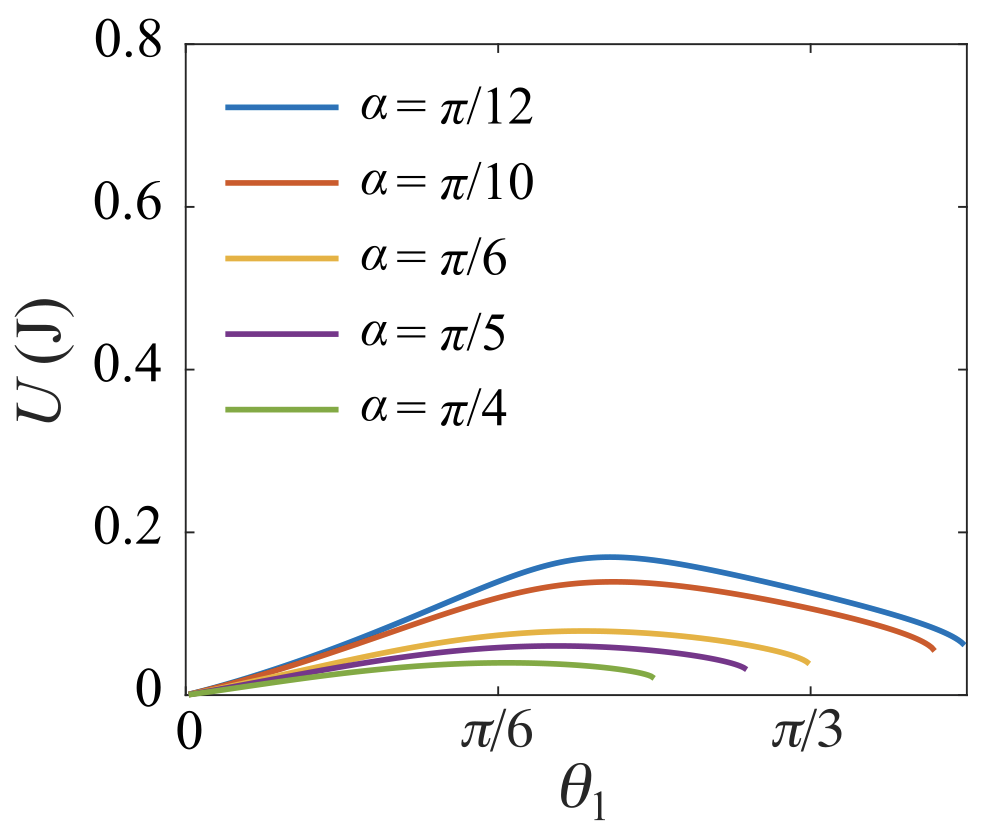


(c) Angle *α*

Figure S6 The elastic energy as a function of the rotational angle *θ*_1_ with different hinge and angle parameters. The initial values of these parameters are: *t_i_* =0.7mm (*i*=1,2,3), *α* = π/6.

Another parameter that has influence on the bistability is the star arm angle *α.* The smaller *α* is, the distance between the star arms is shorter, indicating the deformation of hinge 2 becomes larger. Figure S6(c) shows the comparison of the elastic energy of patterns with different star arm angles. Its tuning capability is more moderate than the hinge width *t*_2_. The smaller *α* is, the more bistable the pattern becomes. This angle will also constrain the working space of *θ*_1_ according to Eq (S26).

**Energy analysis: Finite element simulations**

We build the finite element model to gain further insight into the influence of *t*_1_ on the bistability of the triangle model (Fig.S7a-b). We set the value of *t*_1_/*l_t_* =0.05, 0.25 and 0.5 (there is no vertical cut between two adjacent unit cells). All other hinges have the same thickness *t*/*l_t_* =0.034. When *t*_1_ reaches the maximum, the cuts at the two sides of the unit cell disappear. This design constrains directly the deformation of the four triangles at the corner, which makes the bistability of the pattern reach the extremum. On the other hand, if *t*_1_ is as short as *t*, the structure will not have a second stable equilibrium state. A balance in choosing *t*_1_ and *t* is crucial to realize the structural bistability.

For the star model, the angle of the star arm *β* is tuned for different values and the results are plotted in Fig. S7c-d. The angle does not influence the elastic energy at the second stable state; instead, it determines the energy difference of the highest elastic energy and the energy at the stable state. A smaller *β* leads to a bigger difference, indicating the structure is snappier. Moreover, *β* determines the strain when the bistability takes space. The smaller *β* is, the bigger the strain is, and the structure can deploy to a greater extent.

The metamaterial also has a semi-open stable state because the structure is geometrically compatible at this configuration. This is evident when we apply the load in two steps along each diagonal lines. As shown in Fig. S8a, the red arrow shows the loading direction in each step. In the first step, the pattern opens symmetrically along the diagonal line, and two pairs of triangle opens while the other pairs collapse. When the two pairs of triangles reach the open state, we apply the load along the other diagonal line, which causes the rest pairs of triangles to deploy. Eventually, every pair is fully open. The elastic energy in Fig. S8b indicates the pattern has two stable states, and the resistance force of two steps are shown in Figure S8(c-d).


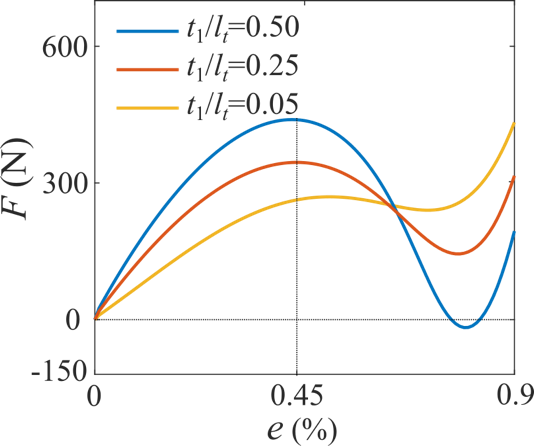

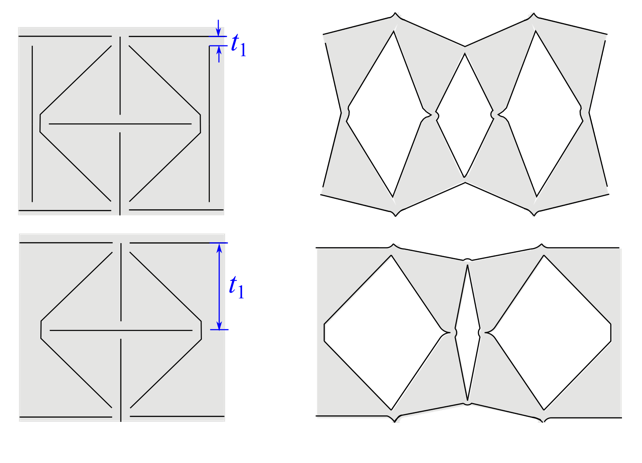


(a) The tuned parameters (b) *t*


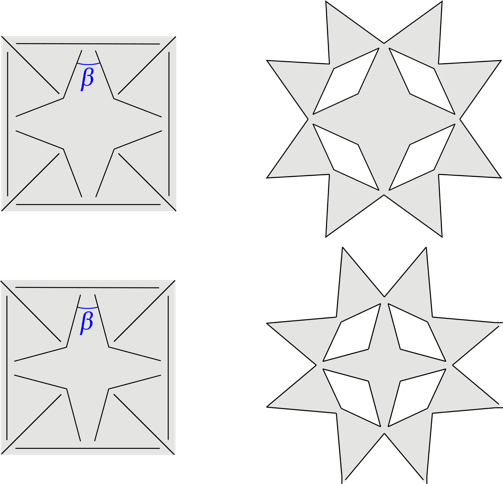

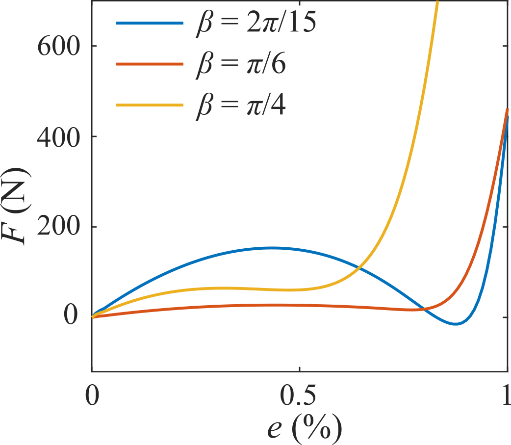


(c) The tuned parameters (d)* β*

**Figure S7 Bistability behaviour of unit cells obtained from FE simulations.**


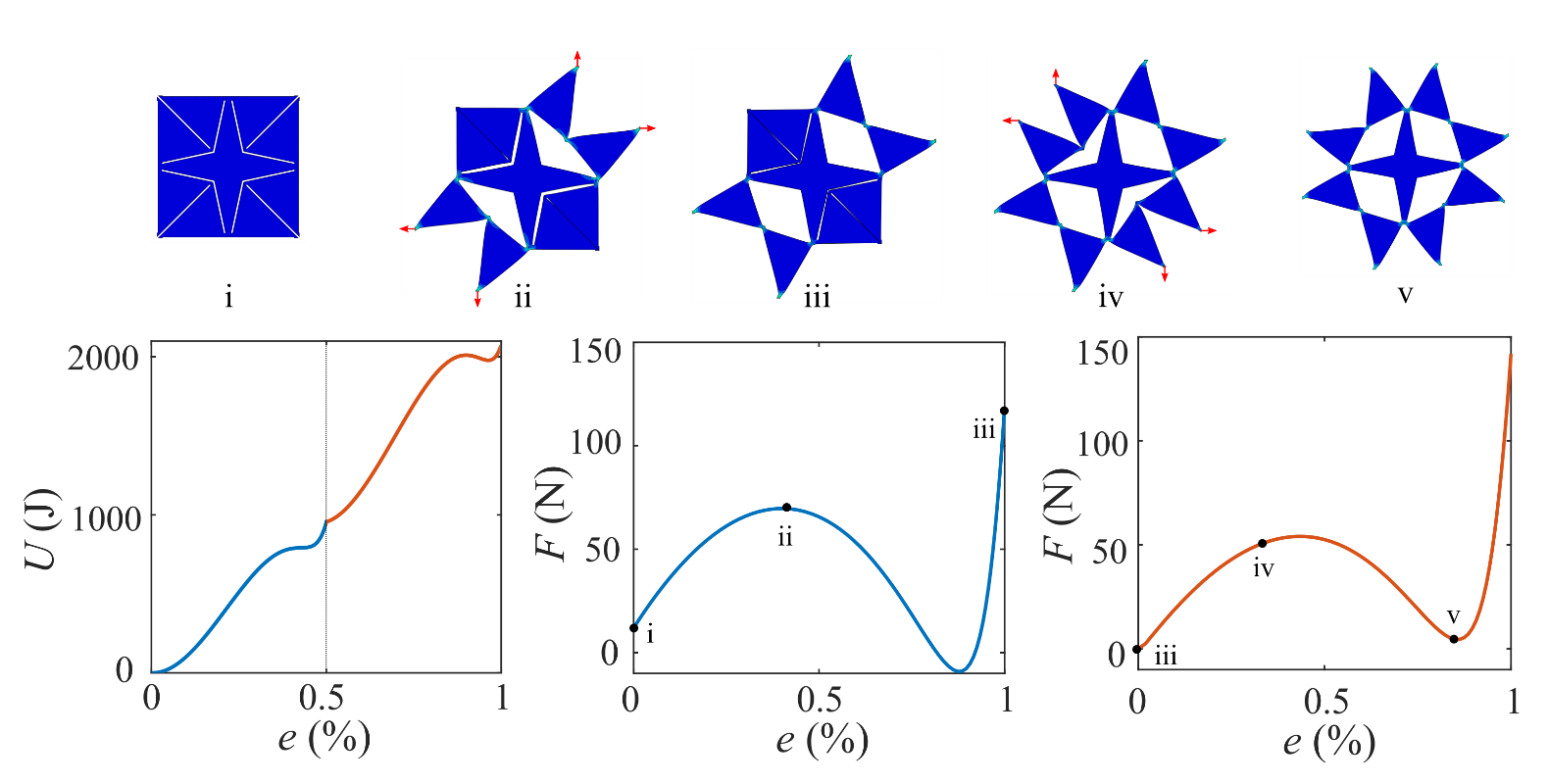


(a) Two step deployment of the star pattern


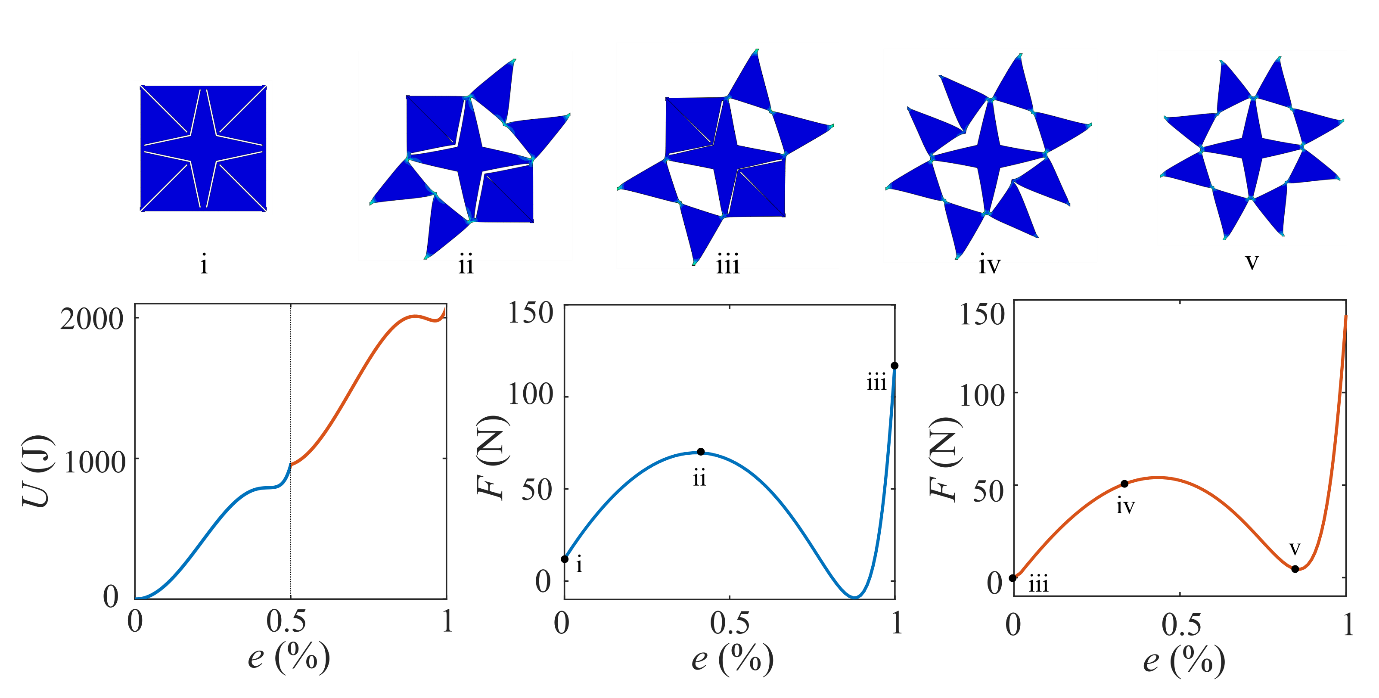


(b) Elastic energy (c) Step 1 (d) Step 2

**Figure S8 Analysis of the semi-stable deformation of the star pattern.**

**Tensile tests**

We fabricated the triangle model specimens with 2×2 unit cells. The unit cell has a dimension of 12mm and there are 8mm of extra rubber at both ends of the specimen so as to be fixed on the frame(Figure S9(a)). The force was measured during loading with a displacement speed of 1 mm/min. We tune the value of *t*_1_ and *t* to observe the bistability of the structure. The results are shown in Figure S9(b).

From Figure S9(b), we can note when *t*_1_/*l* is 0.25 and 0.5, the structure reaches negative force at the second stable states; when *t*_1_ is 0.06 the force is always positive. On the other hand, the influence of *t* is very obvious in changing the strain energy of the structure. If the value of *t*/*l* is big enough, the structure will not be bistable because it cost too much energy to stay in the second stable state.


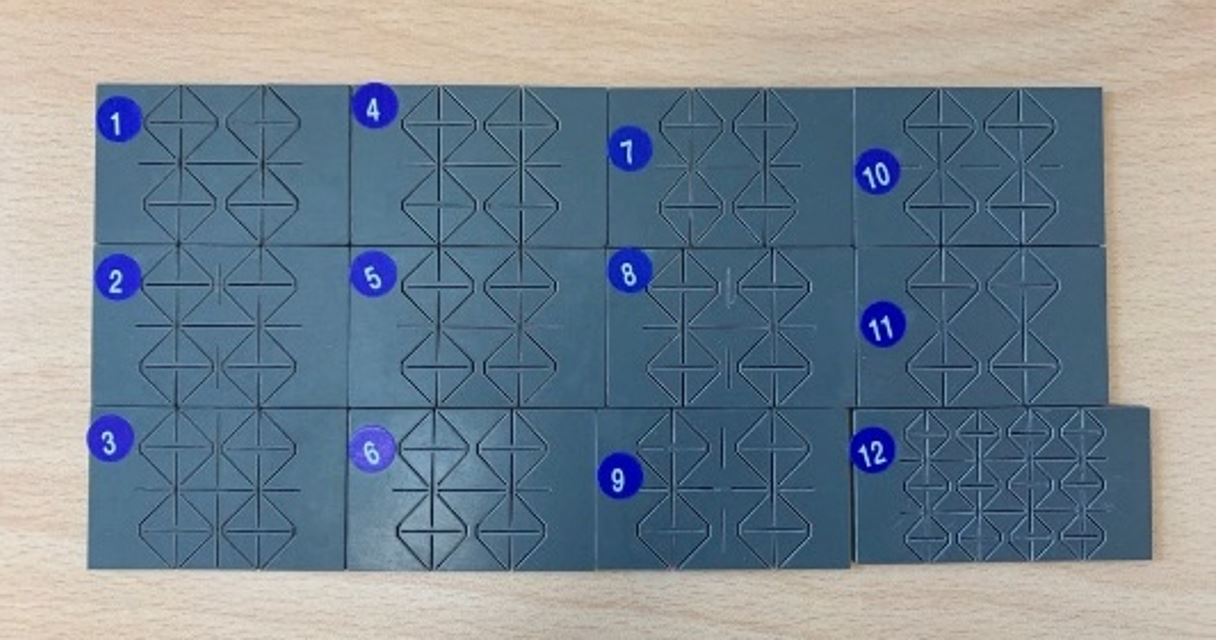


(a) Samples


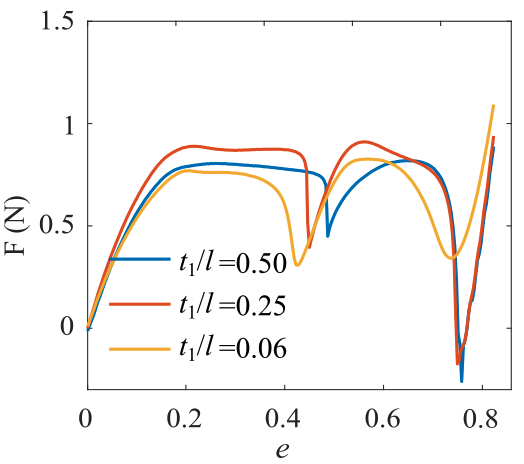

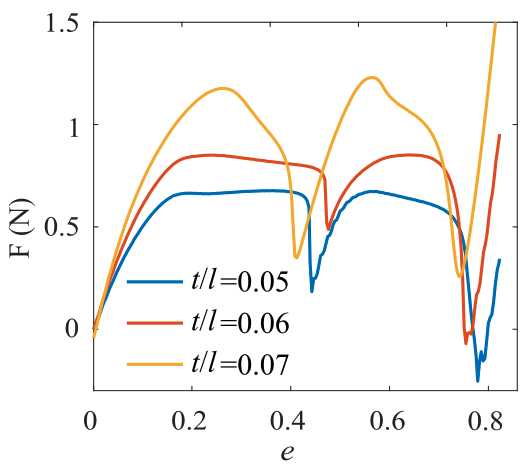


(b) *t*_1_ (c) *t*

**Figure S9 Mechanical response of triangle pattern specimens with different parameters.**

Because of the gravity, the top column will deploy first and generate the first local minima of force. The results will also be influenced by out-of-plane deformation: it has been observed that the more out-of-plane deformation happens, the more difference will there be between the two local minima. The out-of-plane behaviour is undesirable because it causes more deformation in the hinges, which accelerate the speed to failure. We can optimize the experiment set up by adding an acrylic board for guiding lateral expansion. In conclusion, the tensile test results generally agree with the FE simulations and the simplified energy model.

Five star patterns are fabricated with 3×3 unit cells. The dimension of the unit cell is 14mm. A special fixer is designed to hold the sample during the tensile test, as shown in Figure S10(b). Four pieces of acrylic boards with guiding rails are cut and connected to the Instron by 3D printed adapter. The sample is held in between by metal pins that slide on the rail. During the tensile test, the force is evenly transmitted to the metal pins, which expand the sample from four corners in perpendicular directions. We tune the value of s_2_ and *β* as shown in Fig. S10(a). The results are shown in Fig. S10(c)-(d).

According to Fig. S10(c), the thicker the hinge is, the more energy the structure requires to deploy. We can notice when *s*_2_/*l_s_* is 0.044, there are four main drops of forces during the test. The last drop is the biggest because every unit cell reaches its second stable state at this point, while some of them might not be fully deployed in the previous process. The structure can free stand by this configuration. When *s*_2_/*l_s_* increases, the force largely raises to deploy the structure to its second stable state, so the sample can no longer free stand at this configuration. Figure S10(d) shows the influence of *β* on the mechanical response. In accordance with our theoretical results, the smaller *β* is, the more bistable the pattern becomes. In addition, the burr in the curves is generated due to the pins sliding on the guiding rails. This can hardly be prevented because we need a special setup to deploy the sample by stretching four corners in different directions.


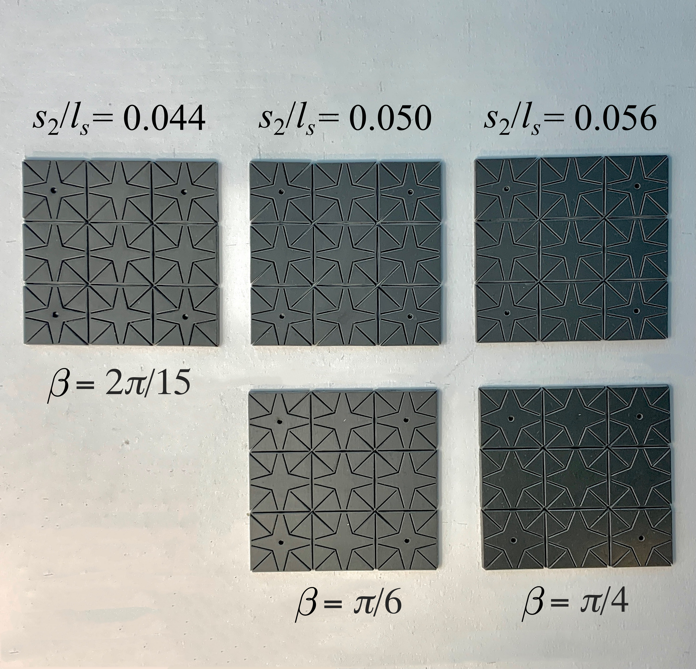

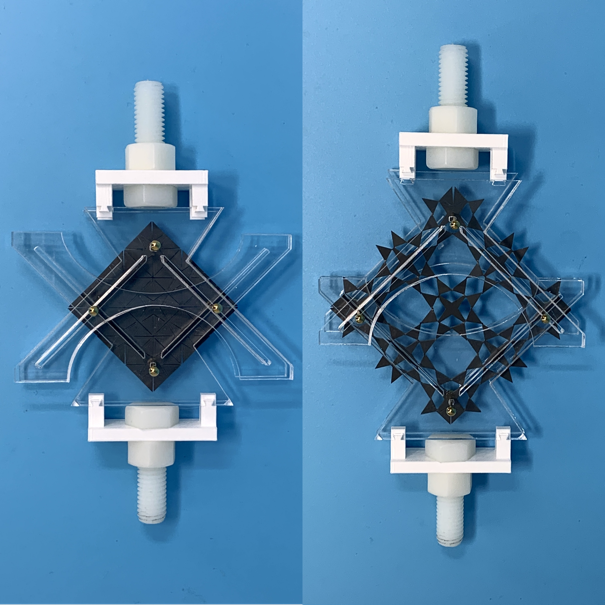


(a) Samples (b) Test setup


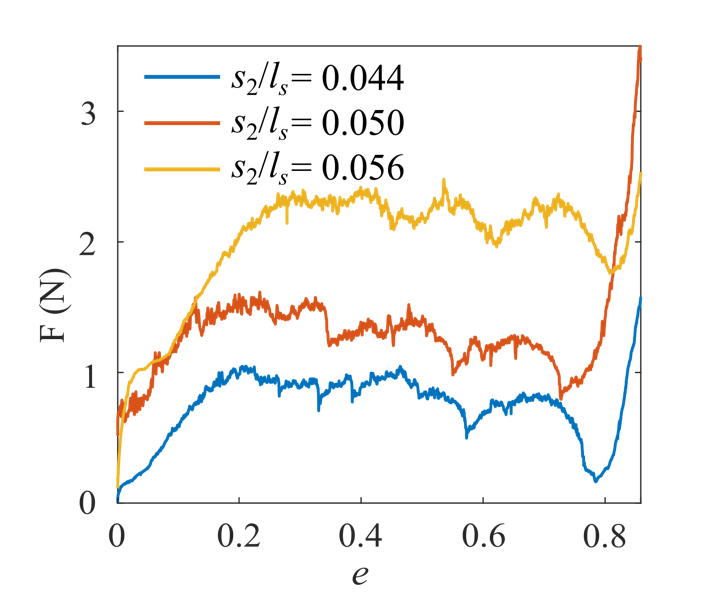

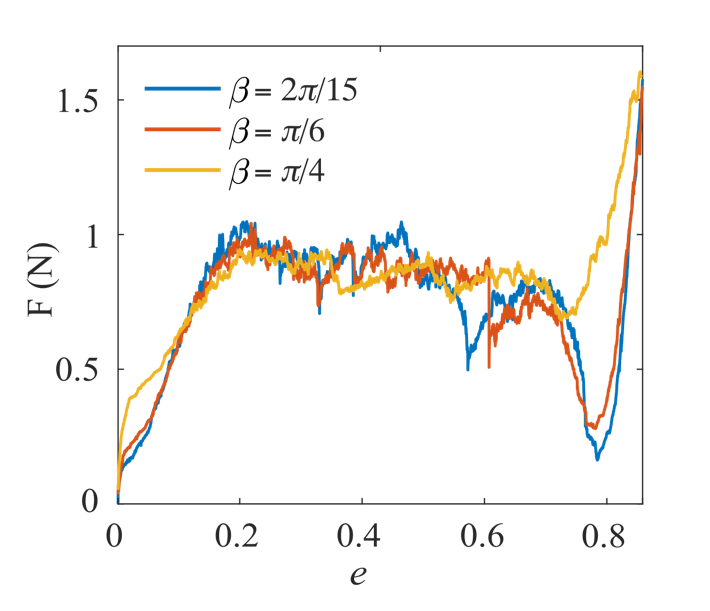


(c) *s*_2_ (d) *β*

**Figure S10 Mechanical response of star pattern specimens with different parameters.**

We deploy the samples for both model to their open state in different speeds to test their reconfiguration repetition. Table 1 shows the time of deployment and the final configuration of the sample. For the triangle sample with 5×5 unit cells, the final configurations remain the same in deployment time of 2s, 5s and 8s, which shows good consistency in repetition. For the star sample with 5×5 unit cells, there is a defect at one corner when the deployment time is 2s. When deployment time is 5s or 8s, the final configurations keep good consistency.

Table 1 Deployment test of metamaterials

| Pattern | Deployment Time | Repetitive Time | Final Configuration |
| --- | --- | --- | --- |
| 3×3 Triangle Pattern | 2s | 3 | 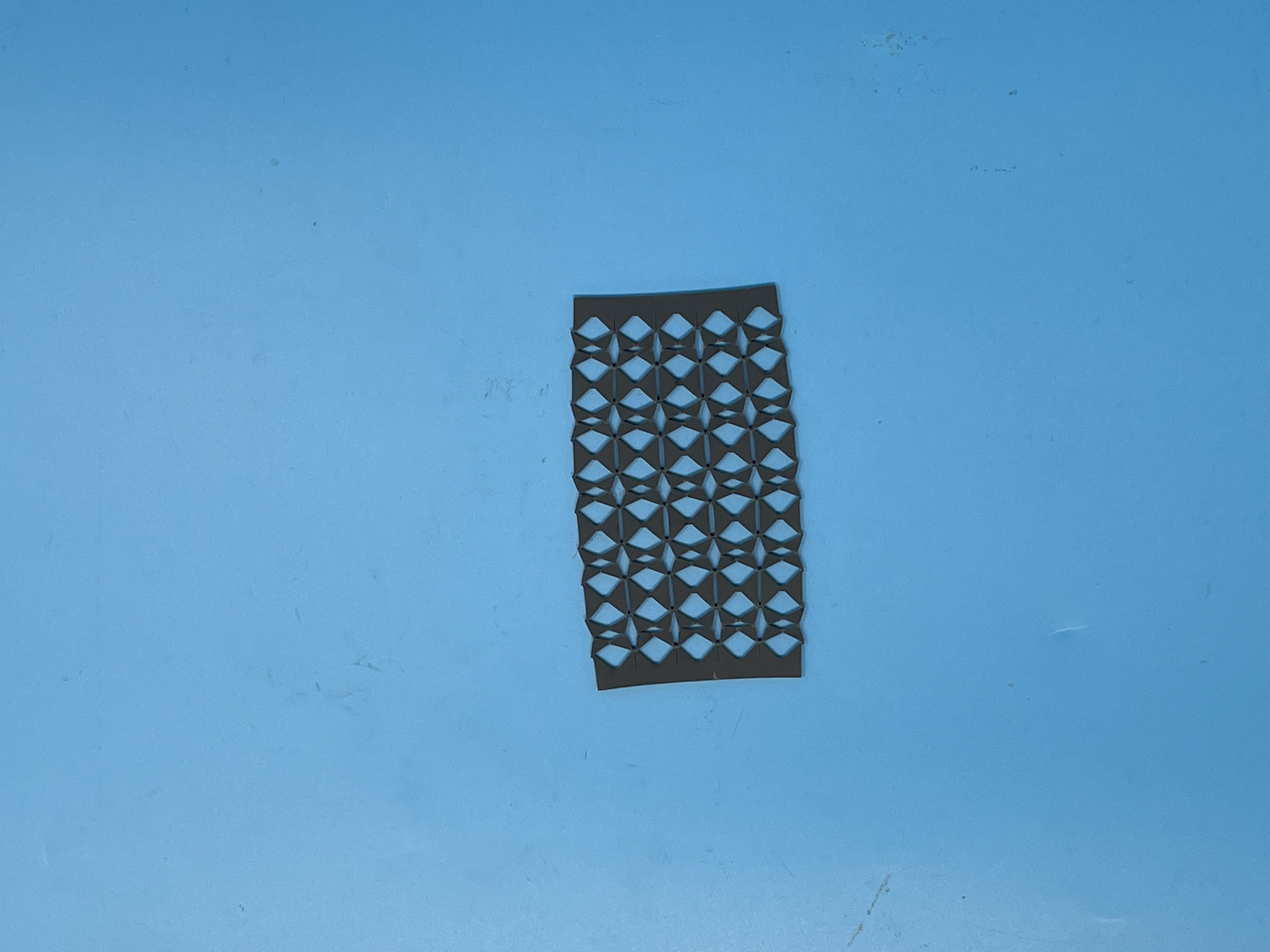 |
|  | 5s | 3 | 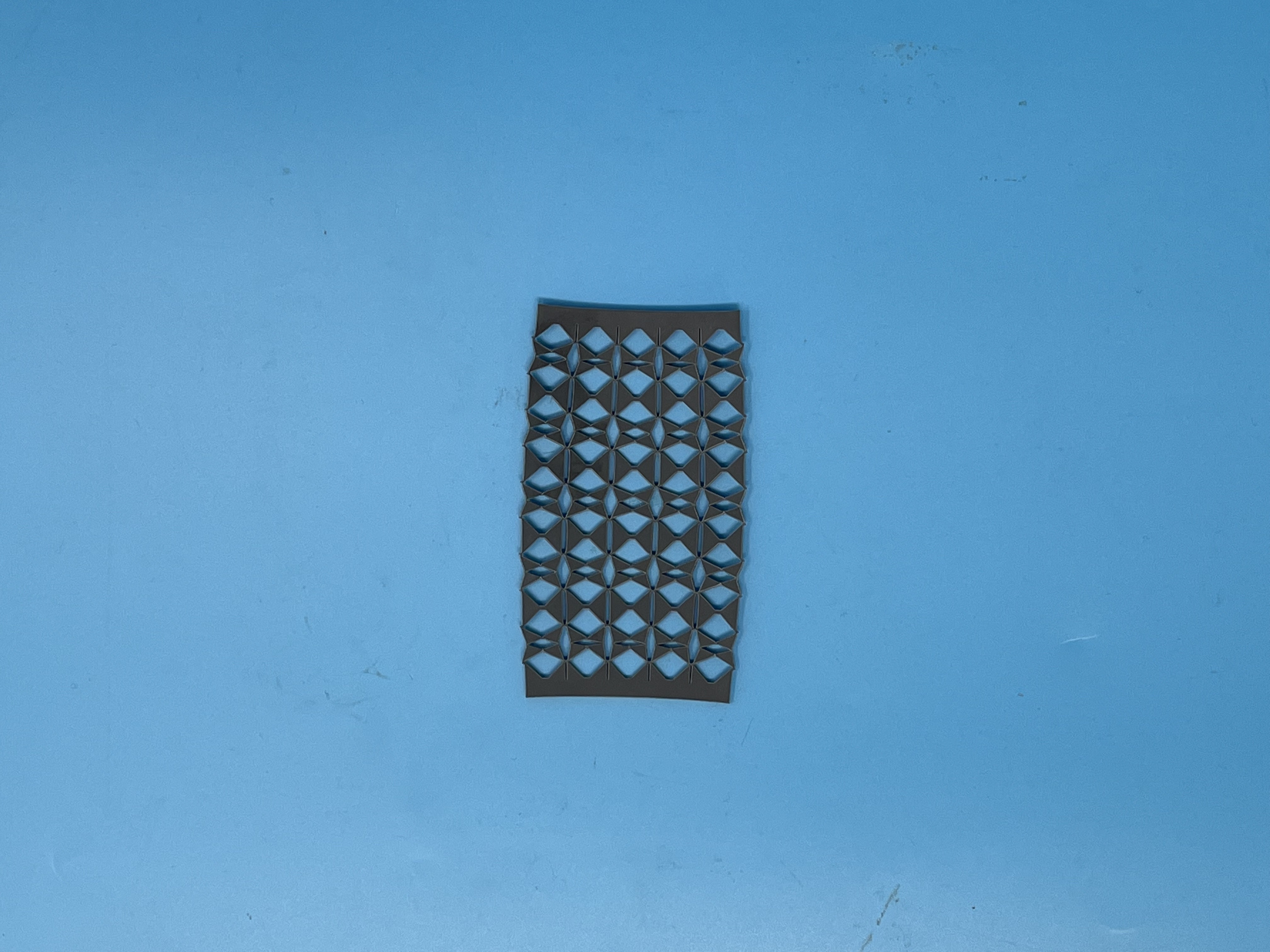 |
|  | 8s | 3 | 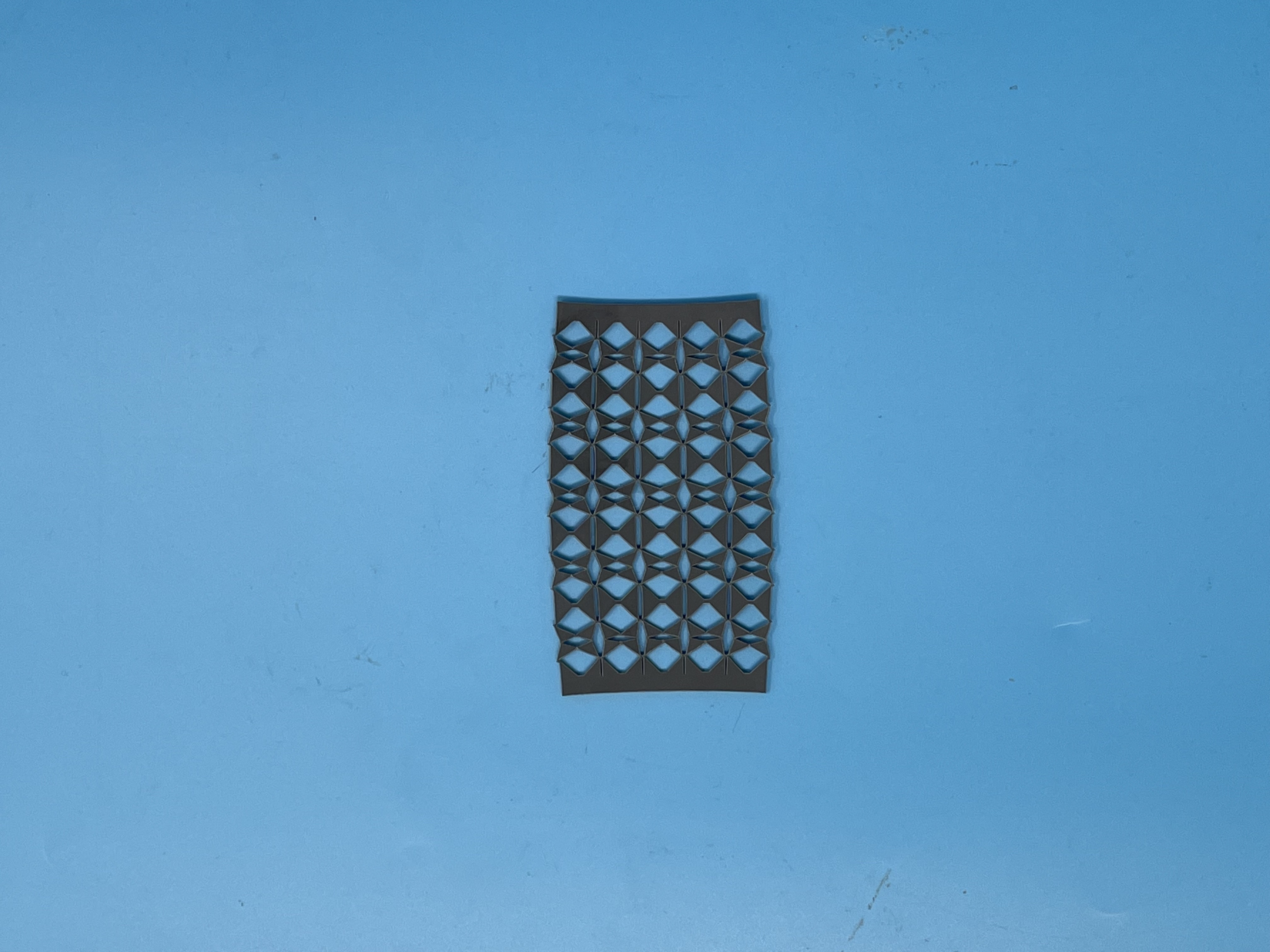 |
| 5×5 Star Pattern | 2s | 3 | 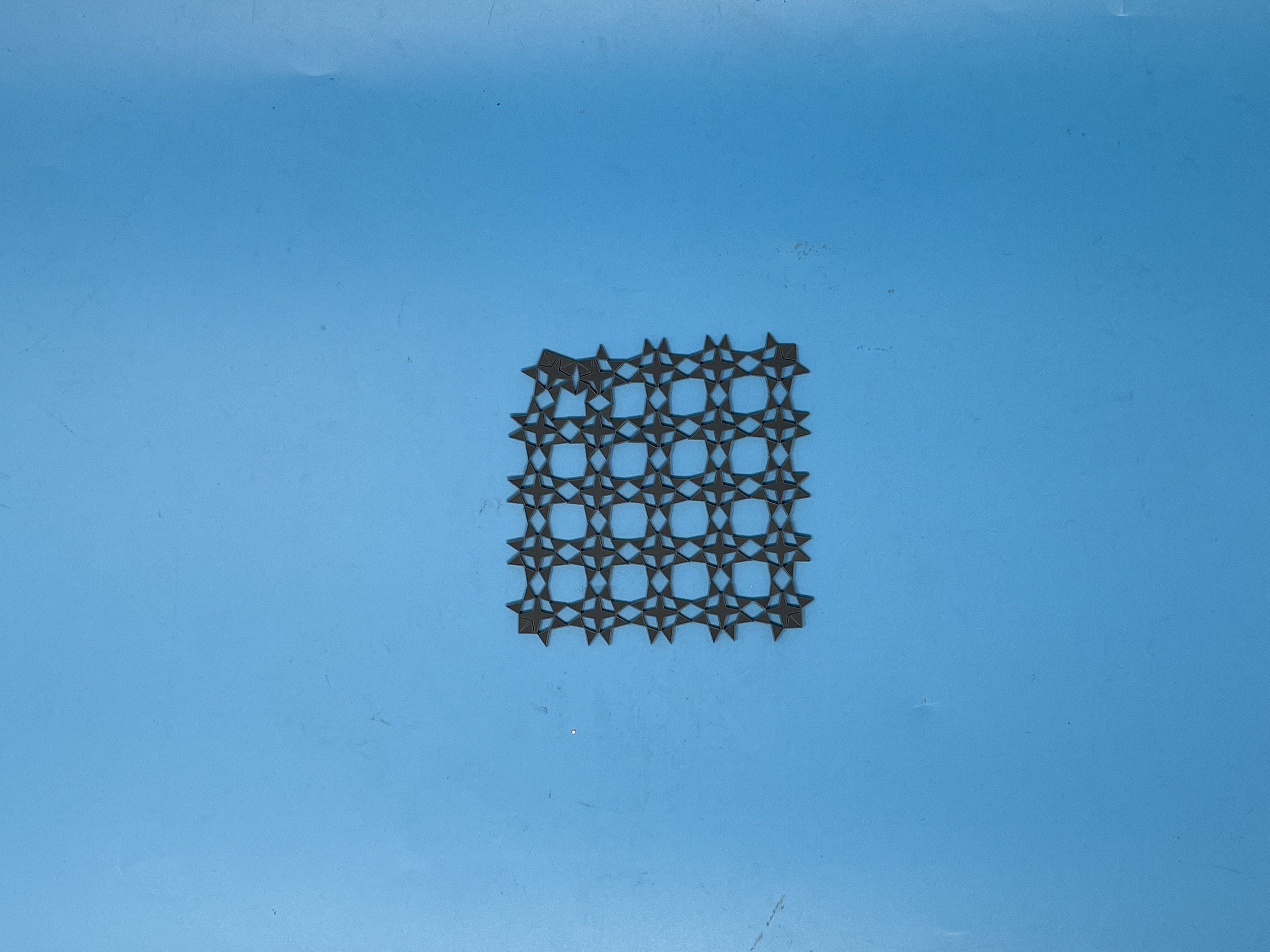 |
|  | 5s | 3 | 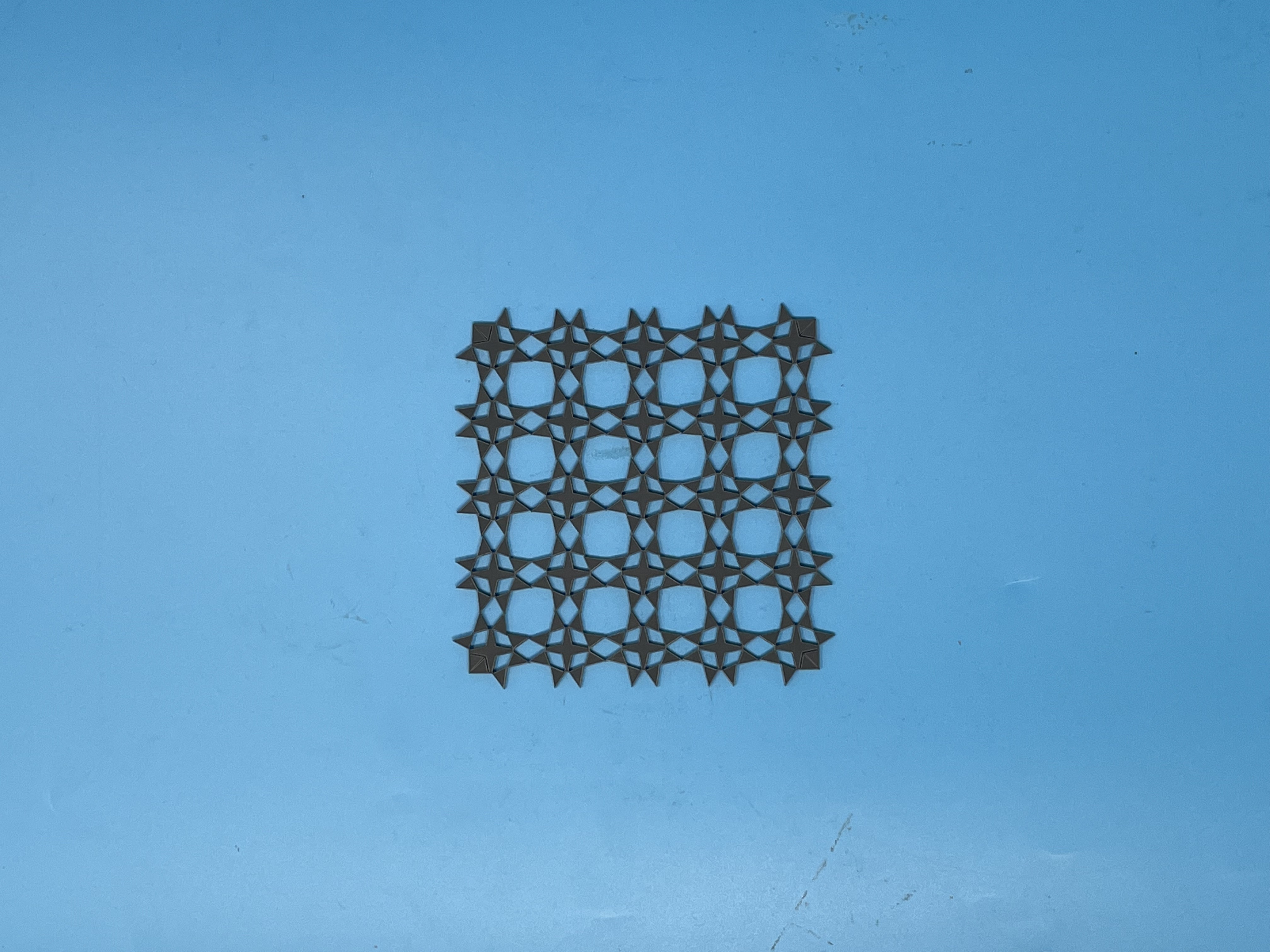 |
|  | 8s | 3 | 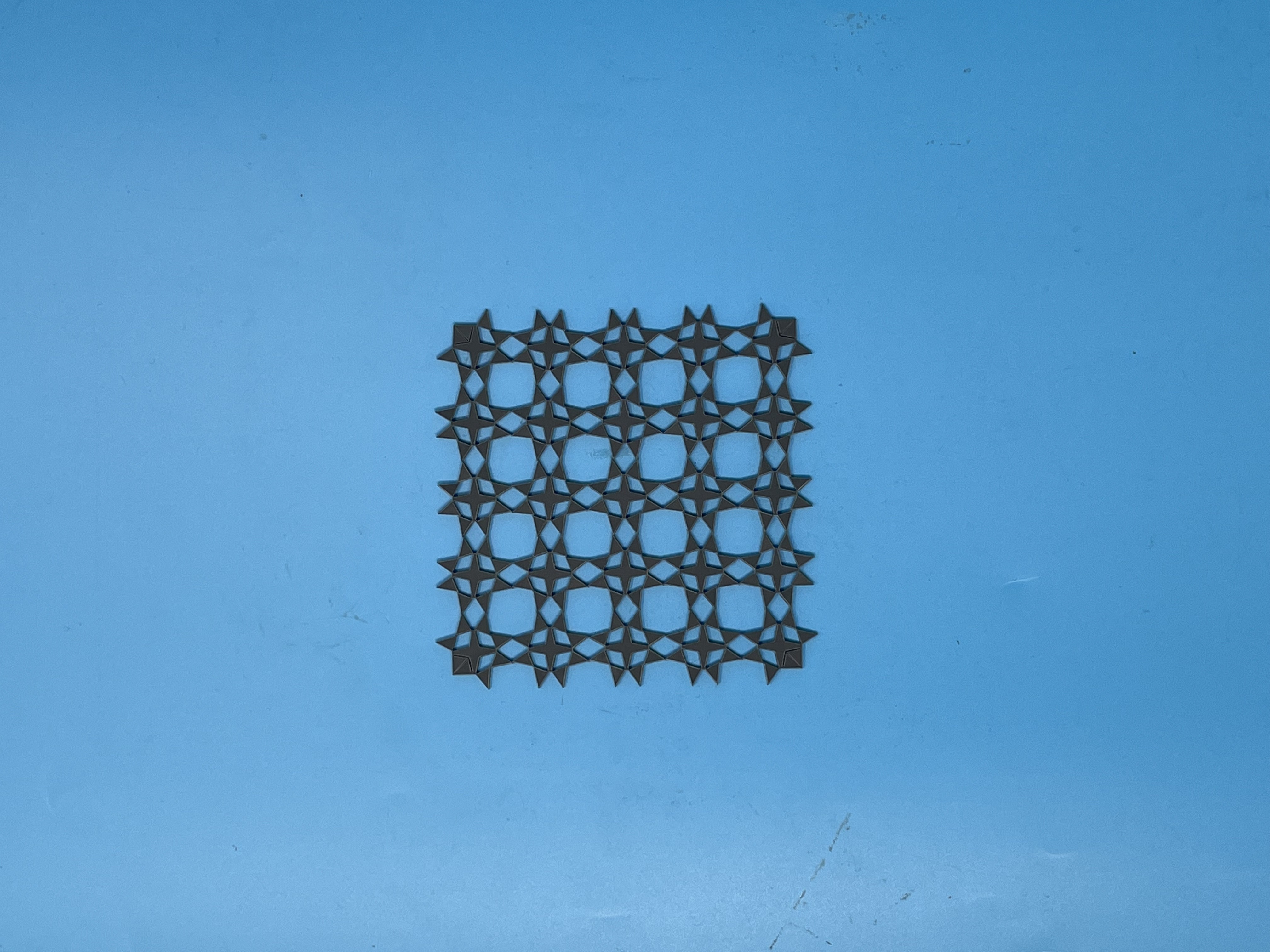 |

Next, we take a closer look at large scale models. For a triangle sample with 10×10 unit cells shown in Fig.S11, the rows of the metamaterial deploy one after the other (Fig. S11a). When the deployment time is 5s, all of the rows deploy successfully and the final configurations is shown in Fig. S11b. This process is repeated for 5 times and the final configurations remain good consistency.


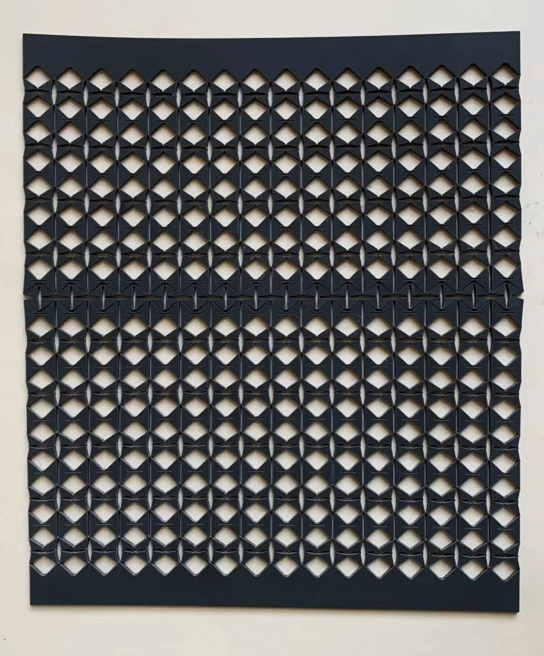

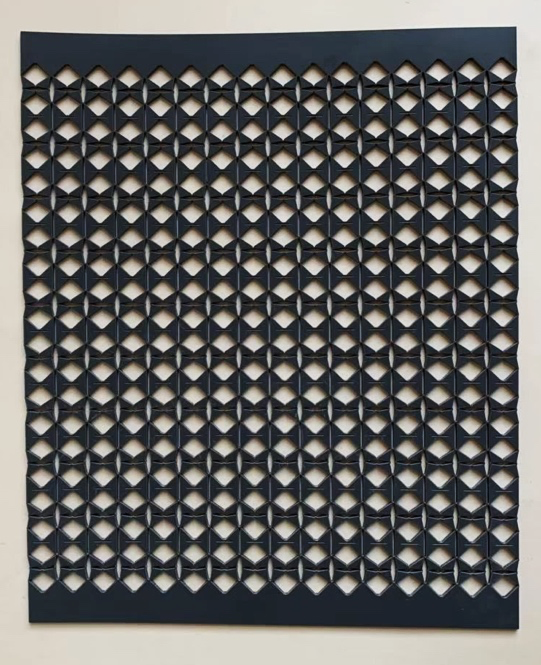


(a) (b)

**Figure S11 Deployment of the large scale triangle metamaterial**

For the star model in large scale, the metamaterial goes through a transition process via manual deployment. As shown in Fig. S12, by pulling the diagonally corner, the metamaterial first deploys to a rhombus shape. Then by pulling the other pair of diagonally corner, the metamaterial can reach the final configuration. This process takes around 8s.


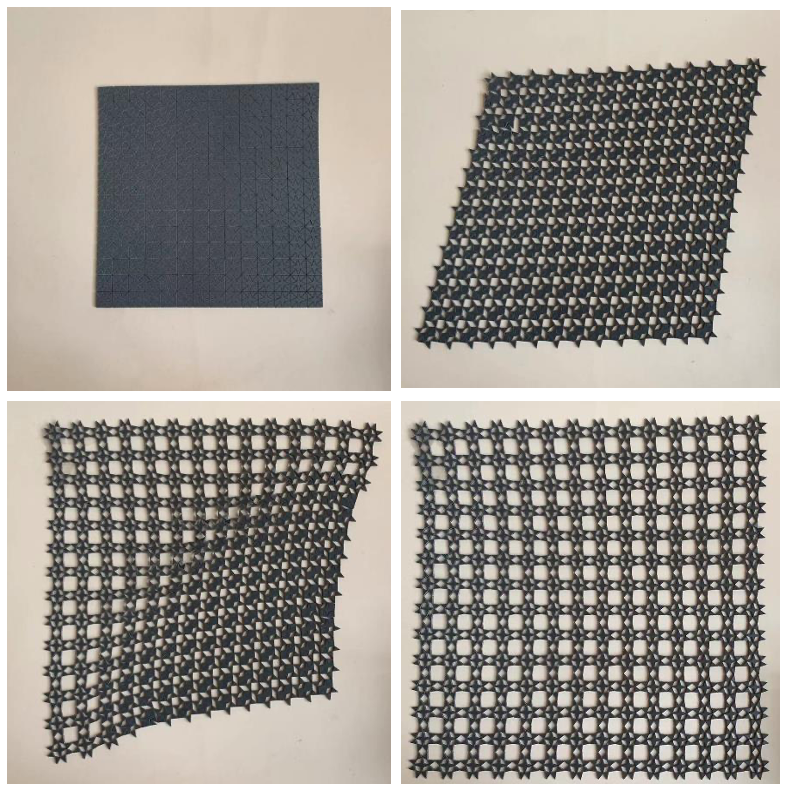


**Figure S12 Deployment of the large scale star metamaterial**

To optimize the deployment process and control the sample uniformly, a control kit, similar to the special fixer mentioned in tensile test section is developed. As shown in Fig. S13, the sample is held in between the acrylic boards by metal pins that slide on the rail of the boards. During the deployment, we pull the adaptors to opposite direction, and this force is evenly transmitted to the metal pins, which expand the sample from four corners in perpendicular directions. Therefore, the star pattern metamaterial can be deployed in one step. This process is also tested for 5 times to validate the consistency of final configurations.


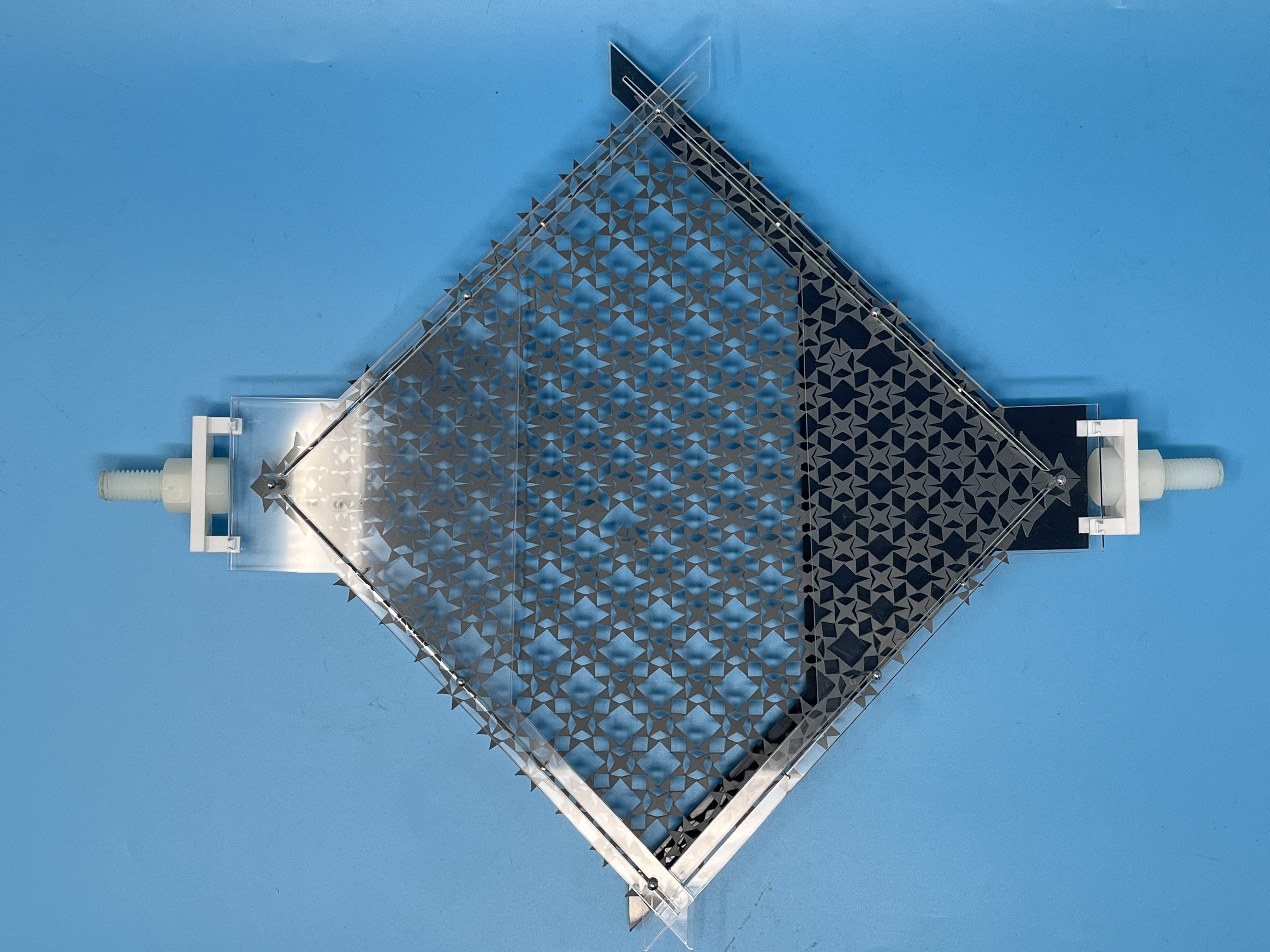


**Figure S13 Deployment of the star metamaterial by control kit**

**Reconfigurable frequency selective surfaces**

Additional numerical results are provided for the reconfigurable frequency selective surfaces (FSSs) created by directly adding a metallic layer on top of the kirigami triangular and star periodic patterns, as discussed in Section 2.4. In particular, we show the absorption responses and the influence on the FSSs response of varying the triangle and star opening angles are investigated numerically through simulations with CST MWS. The geometrical parameters can have a different impact on the positions of the resonances of the FSSs in their open and closed configurations, and thus can be exploited in the design to satisfy specific reconfigurability requirements.

**Triangular kirigami FSS**

The absorption response of the triangle kirigami FSS, whose geometry and transmittance are presented in Figure 4 of the main manuscript, is shown in Figure S14 for both the case of the closed and fully open FSS configurations. It can be noted that absorption can become significant in the vicinity of the FSS resonances, as a result of the losses in the FR4 substrate used for the circuits. While so far in this work, we have prioritised the use of readily available materials with low cost, a wide range of material and design optimizations present themselves, as discussed in the conclusions of our paper, since as more and more composite and other suitable materials become available, the design space becomes increasingly large, providing also the opportunity to significantly mitigate resonance losses through the adoption of low loss supporting substrates.

**
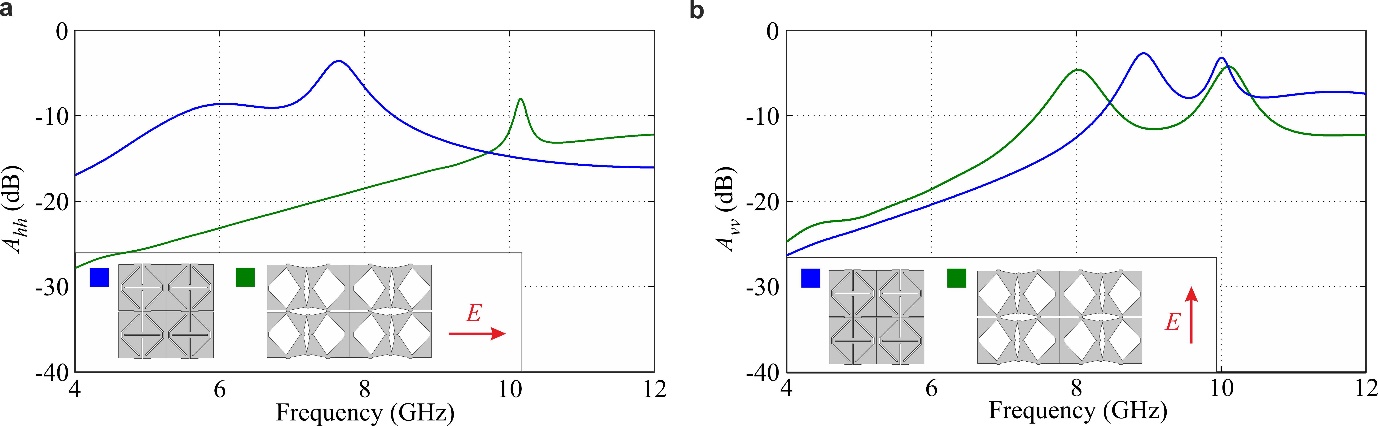
**

**Figure S14 Simulated absorption of the triangular kirigami FSSs with the same unit cell as in Figure 4 of the main manuscript. Absorptions in the closed and fully open configurations for both horizontal and vertical polarizations are compared.**

The transmittances of the triangle kirigami FSSs with variable opening angles $\alpha=30^{\circ},38^{\circ},45^{\circ}$ , as simulated with CST MWS, are shown in Figure S15.

Since the smaller the opening angles, the narrower are the unit cells, the resonances of the FSSs, in both their closed and open configurations and for both incident polarizations, progressively shift downwards in frequency for increasing opening angle. This is not the only effect. For the case of vertical polarized waves, the relative positions of the fundamental resonances of the closed and open FSS configurations drift towards a slightly larger spacing between them and this also affect the level of maximum transmission. Instead, when the incident wave is horizontally polarized, the bandwidth of the first transmission peak appears to progressively reduce while the opening angle of the triangle gets larger. Overall, these results support the conclusion that the opening angle of the triangle unit cell is one of the crucial parameters for the FSS design and need to be carefully selected to achieve the desired reconfigurability requirements.

**
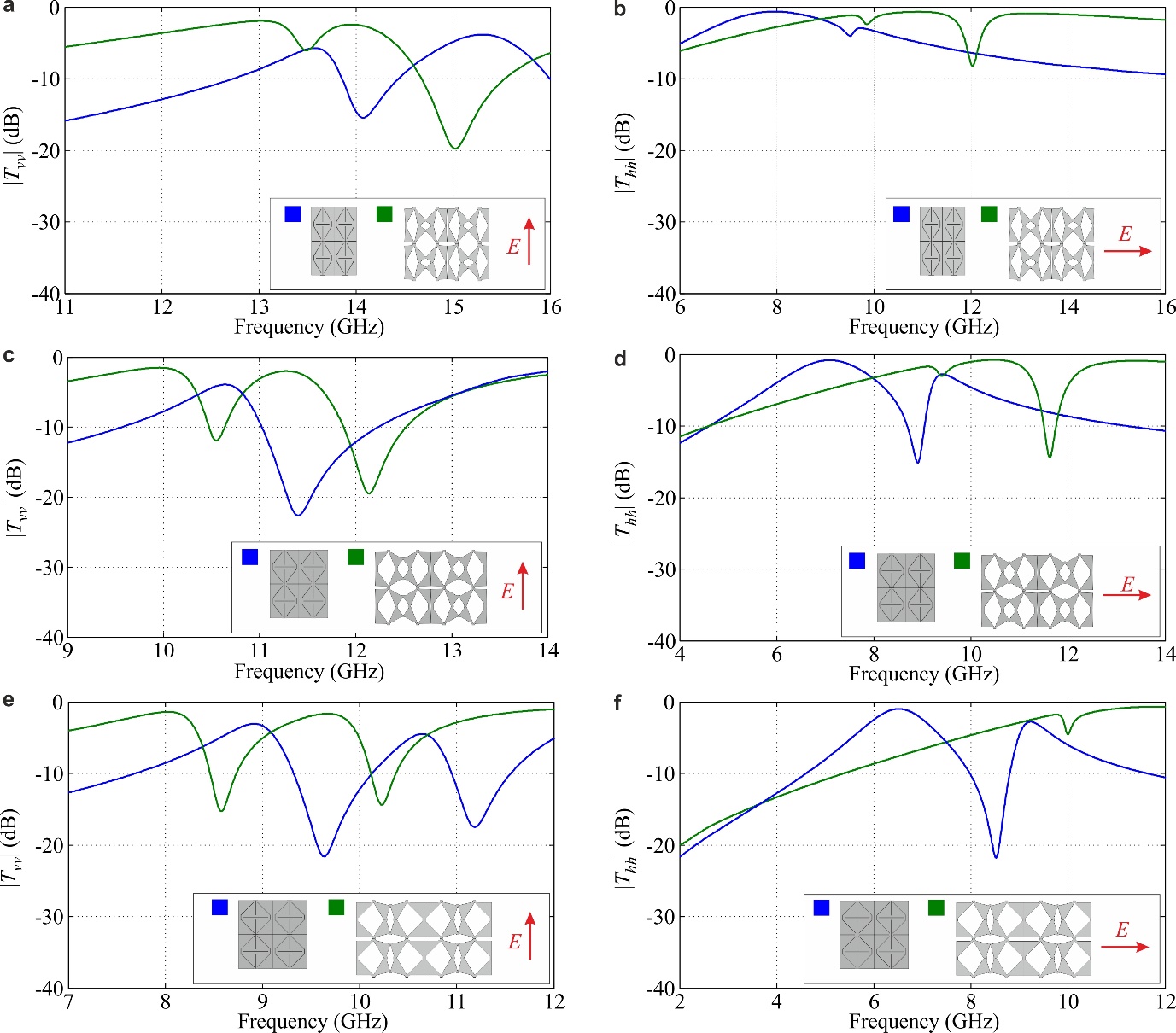
**

**Figure S15 Simulated transmittance of the triangular kirigami FSSs with variable unit cell opening angle α: (a), (b) $\alpha=30^{\circ}$; (c), (d)$\alpha=38^{\circ}$; (e), (f) $\alpha=45^{\circ}.$Transmittance is shown for closed and fully open configurations.**

**Star kirigami FSS**

In Section 2.4.3 we show that a 15% shift in frequency of the FSS fundamental passband response can be obtained by mechanically transforming the kirigami star structure. In the following we demonstrate that the amount of this shift can be also controlled by changing the star opening angle β. Additional simulation results for the transmittance of the star pattern with variable $\beta=30^{\circ},45^{\circ},60^{\circ}$ are presented in Figure S16. These curves show that the FSS response at the fundamental resonance, both in the open and closed states, only slightly shift in frequency for varying β, while a small bandwidth increase can be observed for larger β.


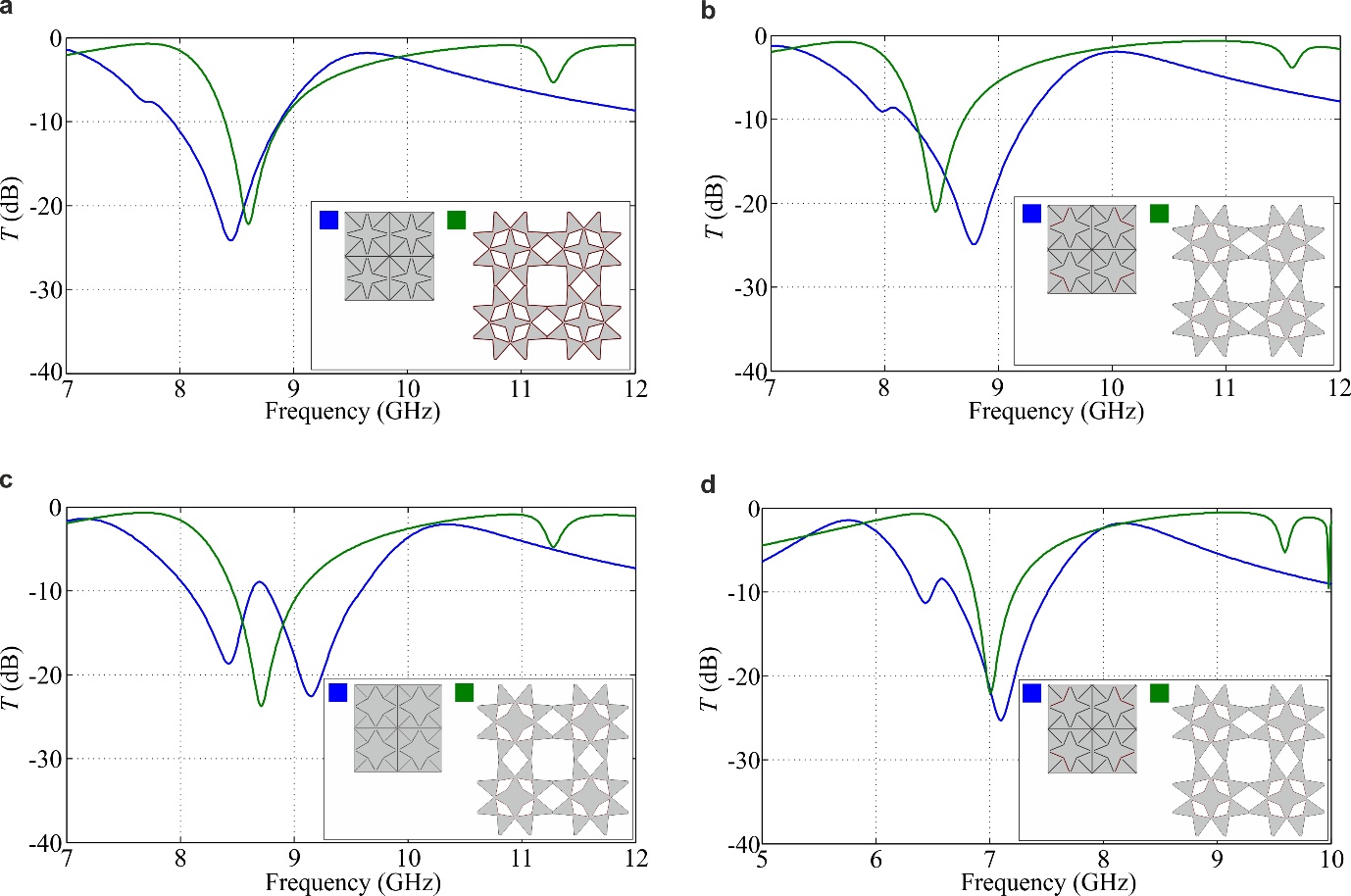


**Figure S16 Simulated transmittance of the star kirigami FSSs with variable opening angle β: (a) $\beta=30^{\circ}$; (b) $\beta=45^{\circ}$; (c) $\beta=60^{\circ}$. (d) Simulated transmittance of the unit cell with $\beta=45^{\circ}$ when size is scaled by a factor 1.25. Transmittance is shown for the closed and fully open configurations.**

However, the trend is not identical for the open and closed FSSs: the response in the open state is more stable with respect to the variation of β; as a result, the shift of the fundamental resonance frequencies of the FSSs when transforming from the open to the closed states and vice versa goes from about 0.55 GHz for the $\beta=30^{\circ}$, to about 0.83 GHz for $\beta=60^{\circ}$. Moreover, at the first stopband, i.e. the first higher order resonance frequency, the response of the FSSs in the closed state changes more significantly with β, while for the open state there is just a slight shift of the corresponding resonance frequency. Generally, the response of the star FSSs in the closed state is affected more noticeably by β, which in turn supports the conclusion that also the width of the cuts outlining the kirigami pattern is another important design parameter.
The last graph in Figure S15(d) shows the simulated transmittance of the unit cell with $\beta=45^{\circ}$ when its size is scaled by a factor 1.25, which as expected simply shift downwards in frequency the responses of the FSS in both its open and closed states.


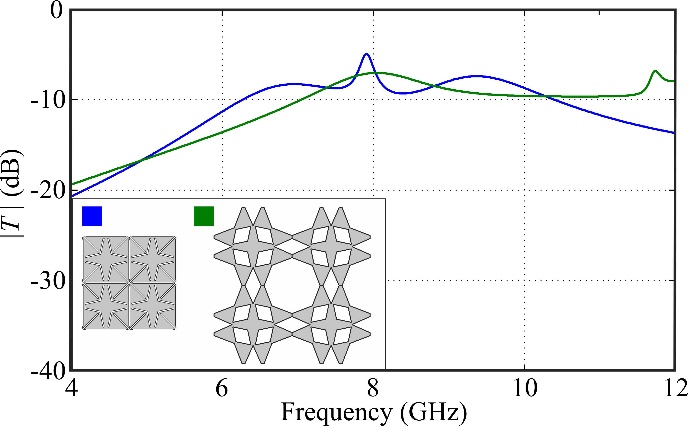


**Figure S17 Simulated absorption of the star kirigami FSS with the same with the same unit cell geometry as that in Figure 4 of the main manuscript. Absorption is shown for both the closed and fully open configurations.**

Finally, in Figure S17, we show the absorption response of the star kirigami FSS, whose geometry and transmittance are presented in Figure 4 of the main manuscript, is shown in Figure S17 for both the case of the closed and fully open FSS configurations. Considerations similar to those developed for the absorption response of the triangular kirigami FSS apply also to these results.
